# Extent of the annual Gulf of Mexico hypoxic zone influences microbial community structure

**DOI:** 10.1371/journal.pone.0209055

**Published:** 2019-04-25

**Authors:** Lauren Gillies Campbell, J. Cameron Thrash, Nancy N. Rabalais, Olivia U. Mason

**Affiliations:** 1 Department of Earth, Ocean, and Atmospheric Science, Florida State University, Tallahassee, FL, United States of America; 2 Department of Biological Sciences, University of Southern California, Los Angeles, CA, United States of America; 3 Department of Oceanography and Coastal Sciences, Louisiana State University, Baton Rouge, LA, United States of America; 4 Louisiana Universities Marine Consortium, Cocodrie, LA, United States of America; Auckland University of Technology, NEW ZEALAND

## Abstract

Rich geochemical datasets generated over the past 30 years have provided fine-scale resolution on the northern Gulf of Mexico (nGOM) coastal hypoxic (≤ 2 mg of O_2_ L^-1^) zone. In contrast, little is known about microbial community structure and activity in the hypoxic zone despite the implication that microbial respiration is responsible for forming low dissolved oxygen (DO) conditions. Here, we hypothesized that the extent of the hypoxic zone is a driver in determining microbial community structure, and in particular, the abundance of ammonia-oxidizing archaea (AOA). Samples collected across the shelf for two consecutive hypoxic seasons in July 2013 and 2014 were analyzed using 16S rRNA gene sequencing, oligotyping, microbial co-occurrence analysis, and quantification of thaumarchaeal 16S rRNA and archaeal ammonia-monooxygenase (*amoA*) genes. In 2014 Thaumarchaeota were enriched and inversely correlated with DO while Cyanobacteria, Acidimicrobiia, and Proteobacteria where more abundant in oxic samples compared to hypoxic. Oligotyping analysis of *Nitrosopumilus* 16S rRNA gene sequences revealed that one oligotype was significantly inversely correlated with DO in both years. Oligotyping analysis revealed single nucleotide variation among all *Nitrosopumilaceae*, including *Nitrosopumilus* 16S rRNA gene sequences, with one oligotype possibly being better adapted to hypoxia. We further provide evidence that in the hypoxic zone of both year 2013 and 2014, low DO concentrations and high Thaumarchaeota abundances influenced microbial co-occurrence patterns. Taken together, the data demonstrated that the extent of hypoxic conditions could potentially drive patterns in microbial community structure, with two years of data revealing the annual nGOM hypoxic zone to be emerging as a low DO adapted AOA hotspot.

## Introduction

Deoxygenation of the ocean is one of the deleterious consequences of global climate change [1,2], with much scientific and public attention directed towards coastal hypoxic zones (dissolved oxygen (DO) concentrations below 2 mg L^-1^ or 62.5 μmol/kg) [3,4]. Hypoxic zones are frequently referred to as “dead zones” because they are inhospitable to macrofauna and megafauna; however, microorganisms thrive in such environments [5]. Eutrophication-associated dead zones have been reported in over 500 locations spanning the globe [6] and are predicted to increase in number and size in the near future as a result of increasing greenhouse gas emissions [2]. The northern Gulf of Mexico (nGOM) is the site of the second largest eutrophication-associated coastal dead zone in the world, with bottom water hypoxia extending to over 20,000 km^2^ [7] and covering anywhere from 20% to 50% of the water column during the summer months [8]. The nGOM hypoxic zone is influenced by stratification and nutrient load from the Mississippi (MS) and Atchafalaya (AR) River freshwater input [9]. Nutrients lead to increased phytoplankton growth, the biomass of which is subsequently respired by aerobic microorganisms, which, combined with poor bottom water ventilation due to strong stratification, leads to low DO concentrations or hypoxic zones [10,11]. Hypoxia in the nGOM has increased in severity during the summer months in direct response to additional inorganic nitrogen loading in the Mississippi watershed, beginning around the 1950s, after which the nitrate flux to the nGOM continental shelf tripled [3,12,13]. The cumulative effects of thermal stratification, nutrient rich freshwater discharge, and microbial respiration culminate and result in an annually extensive nGOM hypoxic zone that reached a record size at 8,776 mi^2^ (22,720 km^2^) in 2017 (LUMCON 2017, https://gulfhypoxia.net/research/shelfwide-cruise/?y=2017, accessed 1/22/2018).

While the sequence of events that leads to the nGOM hypoxic zone is well documented [4,6,12,14,15], efforts to understand how chemical, biological, and physical aspects of hypoxia influence microbial community structure, abundances, and activity across environmental gradients in the coastal shelf have only recently been undertaken [16–20]. King and colleagues [16] reported that before the onset of hypoxia (March), Alpha- and Gamma- Proteobacteria, Bacteriodetes, and Actinobacteria were abundant in nGOM waters < 100 m, with Planctomycetes and Verrucomicrobia being less abundant. We previously sampled during the 2013 hypoxic event in late July, when hypoxic conditions historically and predictably prevail, and reported that the high normalized abundance of a Thaumarchaeota (100% similar to the ammonia-oxidizing archaea (AOA) *Nitrosopumilus maritimus* [21]) Operational Taxonomic Unit (OTU) (16S rRNA gene iTag data), and the absolute abundance of Thaumarchaeota 16S rRNA and archaeal *amoA* gene copies (quantitative PCR (qPCR)) were significantly inversely correlated with DO [19]. In that 2013 study, Thaumarchaeota comprised more than 40% of the microbial community (iTag sequence data) in hypoxic samples [19]. In contrast, King and colleagues [16] and Tolar and colleagues [17] showed Thaumarchaeota generally increased in abundance at depths >100 m in the nGOM when conditions are oxic over the shelf. Bristow and colleagues [18] sampled a similar geographic area in 2012 during a historically small hypoxic zone (smallest recorded since 1988) and reported that thaumarchaeal abundances increased with depth, reaching a maximum at 120 m. However, in their shallow bottom water (15 m) sample, Thaumarchaeota also reached ~15% of the microbial community and the highest rate of ammonia oxidation was reported at this single hypoxic station ([18]; Station 6, see their Fig 3A). These data suggest that nitrification, particularly ammonia oxidation, was actively being carried out in the 2013 hypoxic zone by Thaumarchaeota—an aerobic process that would continue to draw down oxygen, exacerbating hypoxic conditions across the shallow continental shelf [22,23].

In the nGOM hypoxic zone, our understanding of the importance of AOA is only beginning to emerge; however, AOA have been shown to be more abundant than ammonia-oxidizing bacteria (AOB) in a number of terrestrial and marine ecosystems [24–28]. AOA can occupy a variety of marine energetic niches as it has been proposed that AOA can outcompete bacteria in low energy conditions due to their high affinities for ammonia [29] and oxygen [30–32]. Specifically, a culture study by Qin and colleagues revealed active ammonia oxidation and growth of AOA strains under low DO (< 1 μM of O_2_) [30]. In oxygen minimum zones (OMZs), several studies have reported an increase in abundance of Thaumarchaeota in low DO samples [17,32–36]. It has also been observed that the abundance of archaeal *amoA* transcripts increases in OMZs [35,37,38]. Importantly, it has also been shown that Thaumarchaeota can contribute to N_2_O production in oxygen limiting environments, meaning that an abundant AOA population in the nGOM hypoxic zone could be a source of this potent greenhouse gas [30,39,40].

Therefore, to continue to fill in the knowledge gaps on microbial community structure, specifically regarding AOA abundances in the shallow nGOM hypoxic zone (average depth 19 meters below sea level (mbsl)), we followed up on our 2013 survey [19] to describe the microbial communities during July 2014 within and outside of the 13,080 km^2^ hypoxic zone. We further compared these year 2014 (Y14) results to the same thirty-three sites sampled during the year 2013 (Y13) hypoxic zone (15,120 km^2^) [19]. We expanded sampling in Y14 to include surface water and more bottom water OMZ samples compared to Y13. Our goal was to determine whether the extent of hypoxia influences the overall microbial ecology, and in particular the abundance of AOA, across different years. Further, we sought to determine if the high AOA abundances observed in shallow, low DO conditions in this large coastal hypoxic zone occur predictably. If so, this research will contribute to understanding the potential ecological implications for an annual increase of AOA in the nGOM hypoxic zone.

## Material and methods

### Sample collection

The 2014 hypoxic zone was mapped over seven days, from 27 July to 2 August 2014 and measured 13,080 km^2^ (gulfhypoxia.net). At each of the 52 sites, a sample was collected at the surface (1 mbsl; samples designated S for surface; 47 samples total) and near the seafloor at the OMZ (19 mbsl avg. collection depth; samples designated B for bottom; 50 samples total), except for sites A’2, B9, and C7, where surface samples were not taken (Table A in S2 File). Samples were also collected from the surface of the Mississippi River at two sites (0 mbsl; designated R2 and R4 for Mississippi River) for a total of ninety-nine samples. We also determined temperature, depth, salinity (conductivity) and *in situ* chemistry using a SeaBird SBE32 conductivity-temperature-depth (CTD) with a 5L bottle carousel.

### Oxygen, chlorophyll a, and nutrients

Oxygen concentrations were determined *in situ* with the CTD dissolved oxygen sensor and verified using Winkler titrations [41] shipboard. Chlorophyll a samples were concentrated on 25-mm Whatman GF/F filters from 500 ml− ^1^ L seawater and stored at -20°C. Chlorophyll a was extracted using the methods described in the Environmental Protection Agency Method 445.0, “In Vivo Determination of Chlorophyll a in Marine and Freshwater Algae by Fluorescence” [42]; however, no mechanical tissue grinder or HCl was used. Chlorophyll a concentrations were determined using a fluorometer with a chlorophyll a standard (*Anacystis nidulans* chlorophyll a). For nutrients, 60 ml of seawater was filtered through 0.22-μm Sterivex filters into two 30 ml Nalgene bottles and stored at -20°C. Nutrient concentrations were determined by the marine chemistry lab at the University of Washington following the WOCE Hydrographic Program using a Technicon AAII system (http://www.ocean.washington.edu/story/Marine+Chemistry+Laboratory). The sample location map and subsequent plot of DO data were made with Ocean Data View [43].

### Microbial sampling and DNA extractions

From each station, up to 5 L of seawater were collected and filtered with a peristaltic pump both at the surface and at the oxygen minimum (all depths and locations noted in Table A in S2 File). A 2.7 μM Whatman GF/D pre-filter was used and samples were concentrated on 0.22 μM Sterivex filters (EMD Millipore). Sterivex filters were sparged, filled with RNAlater, and frozen. Samples were transported to Florida State University on dry ice and stored at -80°C until DNA extractions and purifications were carried out. DNA was extracted directly off of the filter by placing half of the Sterivex filter in a Lysing matrix E (LME) glass/zirconia/silica beads Tube (MP Biomedicals, Santa Ana, CA, USA) using the protocol described in our previous paper [19], which combines phenol:chloroform:isoamyalcohol (25:24:1) and bead beating. Genomic DNA was purified using a QIAGEN (Valencia, CA, USA) AllPrep DNA/RNA Kit and quantified using a Qubit2.0 Fluorometer (Life Technologies, Grand Island, NY, USA).

### 16S rRNA gene sequencing and analysis

16S rRNA genes were amplified from 10 ng of purified genomic DNA in duplicate using archaeal and bacterial primers 515F and 806R, which target the V4 region of *Escherichia coli* in accordance with the protocol described in [44,45], used by the Earth Microbiome Project (http://www.earthmicrobiome.org/emp-standard-protocols/16s/), with a slight modification: the annealing temperature was modified to 60°C. Duplicate PCRs were combined and purified using Agencourt AMPure XP PCR Purification beads (Beckman Coulter, Indianapolis, IN) and sequenced using the Illumina MiSeq 250 bp, paired-end sequencing. Ninety-nine samples from 52 stations were sequenced and analyzed. All samples in Y13 (39 total samples with 33 samples that were collection from the same stations in Y14) were reanalyzed with the new Y14 samples using the methodology described in detail below for a total of 138 samples collected from both Y13 and Y14. Raw sequences were joined using fastq-join [46] with the *join_paired_ends*.*py* command and then demultiplexed using *split_libraries_fastq*.*py* with the default parameters in QIIME version 1.9.1 [47]. Demultiplexed data matching Phi-X reads were removed using the SMALT 0.7.6 akutils phix_filtering with the *smalt map* command [48]. Chimeras were removed using *vsearch–uchime_denovo* with VSEARCH 1.1.1 [49]. Neither PhiX contamination nor chimeric sequences were observed. Sequences were then clustered into operational taxonomic units (OTUs), which was defined as ≥ 97% 16S rRNA gene sequence similarity, using SUMACLUST [50] and SortMeRNA [51] with *pick_open_reference*.*py -m sortmerna_sumaclust*. Greengenes version 13.5 [52] was used for taxonomy. The resulting OTU table was filtered to keep only OTUs that had 10 sequences or more across all samples (resulting in 8,959 OTUs), and normalized using cumulative sum scaling (CSS) with metagenomeSeq [53] in R. These sequences are available in NCBI’s SRA (accession XXX) and on the Mason server at http://mason.eoas.fsu.edu. While taxonomy was determined using Greengenes, taxonomy for OTUs that were determined to be significantly (Wilcoxon) different between environments (surface and bottom, hypoxic and oxic, Y13 and Y14 samples) were additionally verified beyond the class level using SILVA ACT, version 132 alignment and classification [54] with the confidence threshold set to 70%. To determine close relatives of specific OTUs of interest NCBI’s nucleotide blastn was used to search the Nucleotide collection using Megablast with default parameters [55]. Pairwise sequence comparisons were also carried out using blastn.

### Statistics

Multiple rarefactions and subsequent alpha diversity metrics- Shannon (H’) [56], observed OTUs (calculates the number of distinct OTUs, or richness) and equitability (Shannon diversity/natural log of species richness; the scale is 0–1.0; with 1.0 indicating that all species are equally abundant)- were calculated in QIIME version 1.9.1 using *multiple_rarefactions*.*py* followed by *alpha_diversity*.*py*. The Shapiro-Wilk test (*shapiro*.*test* function) was used to test diversity values and environmental variables for normality in R. In R, the Wilcoxon Rank-Sum test (*wilcox*.*test* function) with the application of Benjamini-Hochberg’s (B-H) False Discovery Rate (FDR) (alpha = 0.05) [57] was used to test for significant differences in diversity values, environmental variables, absolute abundance data (*Thaumarchaeota* 16S rRNA and *amoA* gene copy numbers per L of seawater) and CSS normalized oligotype data between surface and bottom samples, oxic and hypoxic samples, and Y13 and Y14.

Significant differences in all CSS normalized OTU abundances between surface and bottom samples, hypoxic and oxic conditions, and between Y13 and Y14 samples were determined using the non-parametric Wilcoxon test in METAGENassist [58], with the B-H correction for multiple tests. Prior to the Wilcoxon test, data filtering was carried out to remove OTUs that had zero abundance in 50% of samples and the interquantile range estimate was used to filter by variance to detect near constant variables throughout [59]. After this quality filtering, 528 OTUs remained out of 8,959 OTUs for Y14 surface and bottom samples, 623 OTUs remained out of 7,924 OTUs for Y14 bottom only samples, and 724 OTUs remained out of 9,784 OTUs for the same samples collected in Y13 and Y14.

Beta-diversity of CSS normalized data was examined using non-metric multidimensional (NMDS) scaling with Bray-Curtis in R with the *metaMDS* function in the vegan package [60]. The *envfit* function in vegan was then used to fit vectors of environmental parameters onto the ordinations with p-values derived from 999 permutations with the application of the B-H correction for multiple tests using the *p*.*adjust* function (we defined corrected p-values ≤ 0.05 as significant). Using the vegan package in R, the non-parametric test adonis [61] was used to test whether microbial community composition was significantly different between clusters of samples (surface and bottom, hypoxic and oxic, Y13 and Y14, east and west latitudes). The *betadisper* function (vegan) was used to test for homogeneity of multivariate dispersion among sample clusters for surface/bottom samples, hypoxic/oxic samples, Y13/Y14 samples and east/west samples. Betadisper p-values were derived from 999 permutations using the *permutest* function and a B-H correction for multiple tests was performed. Using the psych package [62] in R, the nonparametric Spearman’s rank order correlation coefficient (rho (ρ)) and p-values (B-H correction) were determined for environmental variables and CSS normalized OTUs that were significantly different (Wilcoxon) for Y14 surface and bottom samples, hypoxic and oxic samples, and Y14 and Y13 same samples.

Co-occurrence analysis between OTUs that were significantly (Wilcoxon) different between Y13 and Y14 was carried out by determining Spearman’s correlation coefficients using the psych package in R, similar to [63–66]. Spearman’s correlation results were visualized in R for OTUs that were significantly (corrected p-values ≤ 0.05) correlated with one or more of the three Thaumarchaeota OTUs (4369009, 1584736 and 4073697) that were significantly different between the two years.

### Oligotyping

Shannon entropy (oligotyping) [67,68] analysis was carried out on all 16S rRNA gene sequences identified as *Nitrosopumilus* to identify variability in specific nucleotide positions in this genus. The scripts *q2oligo*.*py* and *stripMeta*.*py* were used to format QIIME generated data for oligotyping [69]. The QIIME command *filter_fasta*.*py* was used to obtain all *Nitrosopumilus* 16S rRNA gene sequences. All *Nitrosopumilus* 16S rRNA gene data was then analyzed using the oligotyping pipeline version 0.6 for Illumina data [67,68] with the following commands, *o-pad-with-gaps*, *entropy-analysis*, and *oligotype*. All oligotype count data was CSS normalized using metagenomeSeq in R. The above analyses were also carried out on all 16S rRNA gene sequences identified as *Nitrosopumilaceae* (excluding those OTUs classified as *Nitrosopumilus* that were already analyzed) to further identify variability in specific nucleotide positions in this family. Combined, these two oligotype analyses encompassed all *Nitrosopumilaceae*, which included the most dominant Thaumarchaeota in our samples.

### Quantitative PCR

Thaumarchaeal and bacterial 16S rRNA and archaeal *amoA* genes were quantified in duplicate using the quantitative polymerase chain reaction (qPCR) assay. For each qPCR reaction 10 ng of genomic DNA was used. Thaumarchaeota 16S rRNA genes were amplified using 334F and 554R with an annealing temperature of 59°C [70]. Bacterial 16S rRNA genes were amplified using 1369F and 1492R with 56°C as the annealing temperature [70]. Archaeal *amoA* genes were amplified using Arch-*amoA*-for and Arch-*amoA*-rev with 58.5°C as the annealing temperature [26]. Standards (DNA cloned from our samples for Thaumarchaeota 16S rRNA and archaeal *amoA* and *E*. *coli* for bacterial 16S rRNA) were linearized, purified, and quantified by fluorometry. The reaction efficiencies for the standard curve were calculated from the slope of the curve for all qPCR assays and were 91.1% for Thaumarchaeota 16S rRNA genes, 88.5% for bacterial 16S rRNA genes and 85.3% for archaeal *amoA* genes. The qPCR data was converted to gene copies L^-1^ of seawater.

### Ethics statement

Permission was not required for sample collection because the sampling sites were not part of a private or protected area. No endangered species were involved in this study.

### Data deposition

These sequences are available at http://mason.eoas.fsu.edu and from NCBI's sequence read archive (BioProject ID: PRJNA532906).

## Results

### In situ chemistry and physical attributes of the 2014 hypoxic zone

All environmental parameters measured, including DO concentrations in all ninety-nine surface and bottom samples and the two surface samples from sites at the MS River mouth (R4 and R2) collected in late July from Y14 are shown in Table A in S2 File. DO ranged from 4.5 mg/L to 13.4 mg/L in surface samples and 0.13 mg/L to 5.73 mg/L in bottom samples. Ammonium (NH_4_) concentrations ranged from 0.2 μM to 2.4 μM in surface samples and 0.3 μM to 6.7 μM in bottom samples, while nitrite plus nitrate (NO_2_ + NO_3_) concentrations ranged from 0.3 μM to 42.5 μM in surface samples and 0.4 μM to 15 μM in bottom samples. Phosphate (PO_4_) concentrations ranged 0.5 μM to 1.8 μM in surface samples and 0.89 μM to 2.9 μM in bottom samples. The average salinity concentration in surface samples was 26.7 ppt and in bottom samples was 35 ppt. The average temperature in surface samples was 29.7°C and the average in bottom samples was 25°C. The average depth of our samples was 19 mbsl. In Y14, all environmental variables (Table A in S2 File) except chlorophyll a were significantly different between surface and bottom samples (Wilcoxon with B-H corrected p-values; Table B in S2 File). Average NO_2_ + NO_3_ concentrations were higher in surface samples compared to bottom samples, while NH_4_ and PO_4_ were higher in bottom samples. River sample R4 had a salinity of 2 ppt and R2 had a salinity of 10.5 ppt. River sample R4 had a combined NO_2_ + NO_3_ concentration of 194 μM and R2 had a combined concentration of 71.7 μM.

Bottom water hypoxic conditions in Y14 were confined to the coast and shallower depths (Figure A in S1 File), reaching a total area of 13,080 km^2^. When looking at bottom samples only, all environmental variables except NH_4_, temperature, and chlorophyll a were significantly different between hypoxic and oxic samples (Table B in S2 File). Average NO_2_+NO_3_ and PO_4_ concentrations were higher in hypoxic water samples compared to oxic samples, while average salinity concentrations were higher in oxic samples. The average depth of the bottom water hypoxic samples was 15 m and the average depth of oxic samples was 25 m.

### Microbial community composition across the shelf and with depth in the 2014 hypoxic zone

**Fig 1:**
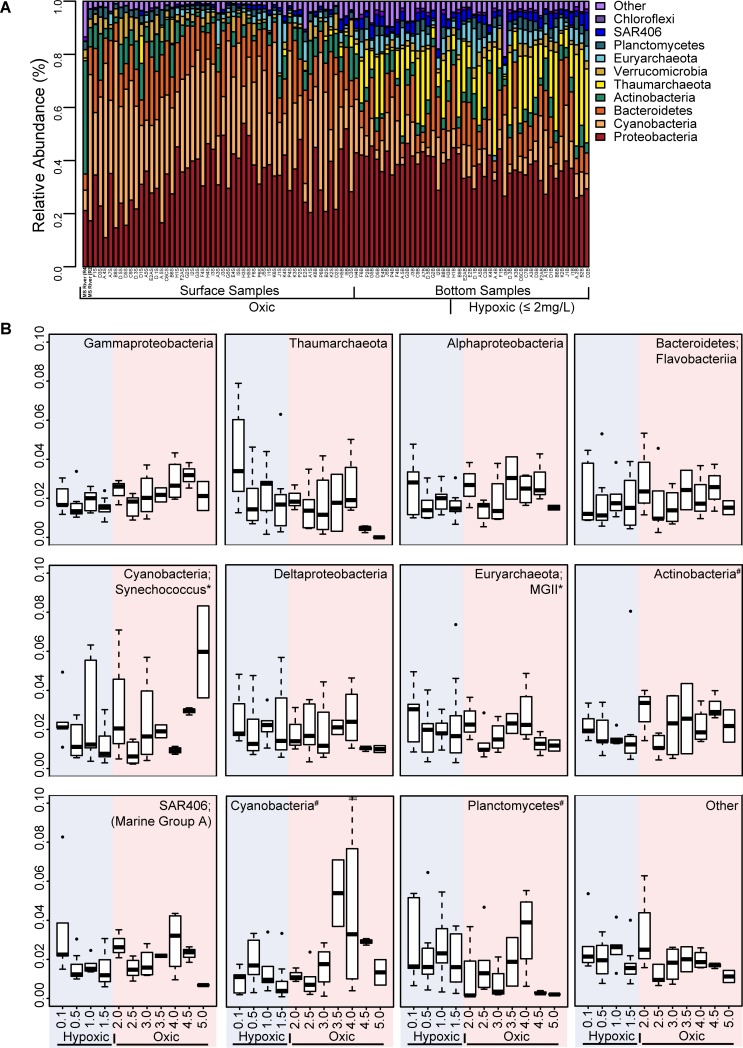
Distribution of abundant bacterial and archaeal phyla. **(A)** Phylum-level bar graph of relativized 16S rRNA gene iTag sequence data, in which only the more abundant bacterial and archaeal groups are shown. Less abundant groups were summed under “Other.” Samples are sorted from lowest to highest DO concentrations on the x-axis and surface and bottom samples are differentiated by brackets with the two Mississippi (MS) River samples on the far left. **(B)** Boxplots of the most abundant groups for bottom samples plotted along a DO gradient from lowest to highest DO concentrations (* indicates that taxonomy for these phyla were further refined based on the OTU taxonomy in these groups. # indicates that taxonomy was not resolved beyond phylum).

iTag sequencing of 16S rRNA genes was used to determine microbial community composition across the shelf in the Y14 nGOM hypoxic zone in surface and bottom samples. This sequencing effort resulted in 11.9 million reads and 8,959 OTUs (the full OTU table is included as Table C in S2 File). In the two MS River samples, Actinobacteria, Cyanobacteria, and Proteobacteria were the most abundant phyla (Fig 1A) (Thaumarchaeota relative abundances were low, with R4 relative abundances being 1.4% and R2 abundances <0.001%). Actinobacteria OTU4345058 (ac1) relative abundance was the highest (up to 22% relative abundance at site R4 and 0.7% at the more saline R2 site), decreasing to 0.1% in surface samples near the mouth of the MS River to < 0.001% to undetectable outside of the MS River in surface and bottom samples (avg. in surface samples 3.54 × 10^−4^ and avg. in bottom samples 3.8 × 10^−5^). Cyanobacteria (*Cyanobium* PCC-6307) OTU404788 was the second most abundant OTU in river samples with a higher abundance in the more saline R2 sample (up to 28% at site R2 and 3% at R4). The surface microbial communities had similar dominant phyla to the MS River samples with Cyanobacteria (avg. 33%) and Proteobacteria (avg. 32%), predominantly *Gamma*- and *Alphaproteobacteria*, being the most abundant, but had lower abundances of Actinobacteria (3%) than in the two river samples (Fig 1A). In surface samples the average relative abundance of Thaumarchaeota, *N*. *maritimus* OTU4369009 was 0.6%.

Bottom water (avg. collection depth 19 mbsl and avg. DO concentration 2.26 mg/L) samples were dominated by Proteobacteria (avg. 37%), primarily *Gamma*- *Alpha*- and *Deltaproteobacteria*, as well as Thaumarchaeota (avg. 14%) (Fig 1A and 1B). The dominant Thaumarchaeota, *N*. *maritimus* OTU4369009, had an average relative abundance of 13% in bottom water samples. When plotting bottom water data along a DO gradient, several trends emerged. *Gammaproteobacteria*, *Alphaproteobacteria*, Bacteroidetes, Cyanobacteria, and the *Acidimicrobiia* generally increased in relative abundance with higher DO (Fig 1B). *Deltaproteobacteria*, MGII Euryarchaeota, and Planctomycetes generally increased in abundance with decreasing DO (Fig 1B). In hypoxic samples, Thaumarchaeota were most abundant, particularly at the lowest DO concentration, with decreasing abundances as DO increased (Fig 1B). Of these taxa in the bottom samples, the normalized abundances of Thaumarchaeota and Planctomycetes were significantly inversely correlated with DO (Spearman’s ρ for Thaumaechaeota = -0.38 and Planctomycetes = -0.35, corrected p-values ≤ 0.05).

### Microbial ecology and correlation analyses with environmental variables across the 2014 hypoxic zone

#### Surface and bottom samples

Statistical analysis of alpha diversity metrics revealed that microbial diversity (Shannon) was significantly lower in the surface samples when compared with bottom samples (Wilcoxon with B-H corrected p-values; Table B in S2 File). Specifically, Shannon diversity indices averaged 5.97 in all nGOM surface samples and 6.02 in the two surface river samples, as compared with 6.45 in bottom samples. Richness (observed species) increased significantly with depth (avg. 329.92 in surface samples and avg. 434.89 in bottom samples). A test of evenness (equitability) between the surface and bottom sample types revealed that surface samples (avg. 0.72) were less even than bottom samples (avg. 0.75).

Non-parametric statistical analysis (Wilcoxon) was then used to determine which OTUs were responsible for the significant difference in species richness when comparing all surface and bottom samples. Seventeen OTUs showed significant differences in their CSS normalized abundances between surface and bottom samples (Figure B in S1 File). Seven of these OTUs had higher average CSS normalized abundances in bottom samples and were significantly inversely correlated with DO (Figure B in S1 File; Spearman’s ρ and corrected p-values ≤ 0.05 in Tables D and E in S2 File). The dominant Thaumarchaeota, *N*. *maritimus* OTU4369009, was significantly inversely correlated with DO and positively correlated with NO_2_ + NO_3_ and PO_4_ (Tables D and E in S2 File).

#### Bottom water samples

When analyzing bottom water samples alone, Shannon diversity, richness, and evenness indices for hypoxic versus oxic samples were not significantly different. Comparison of these same samples (Wilcoxon) revealed that the CSS normalized abundances of 16 OTUs were significantly different depending on DO status (Figure C in S1 File). Of these 16 OTUs, six had higher CSS normalized abundances in hypoxic samples and were significantly inversely correlated with DO (Figure C in S1 File; Spearman’s ρ and corrected p-values ≤ 0.05 in Tables F and G in S2 File). *N*. *maritimus* OTU4369009 comprised an average of 16% of the microbial community in hypoxic samples, with a peak abundance of 33% of the microbial community in sample A’2 bottom, which had one of the lowest DO concentrations (0.31 mg of O_2_ L^-1^), versus an average of 10% relative abundance in oxic samples. Further, the same OTU *N*. *maritimus* OTU4369009 was significantly inversely correlated with DO in Y14 bottom samples and significantly inversely correlated with NH_4_ and temperature in bottom samples (Tables F and G in S2 File). Furthermore, *N*. *maritimus* OTU4369009 was significantly positively correlated with NO_2_+NO_3_, PO_4_, and salinity in bottom samples (Tables F and G in S2 File). While this Thaumarchaeota OTU4369009 was abundant in hypoxic samples and inversely correlated with DO, the normalized abundances were not significantly different between hypoxic and oxic bottom samples (Wilcoxon).

### Microbial community organizational structure and drivers in the 2014 hypoxic zone

**Fig 2:**
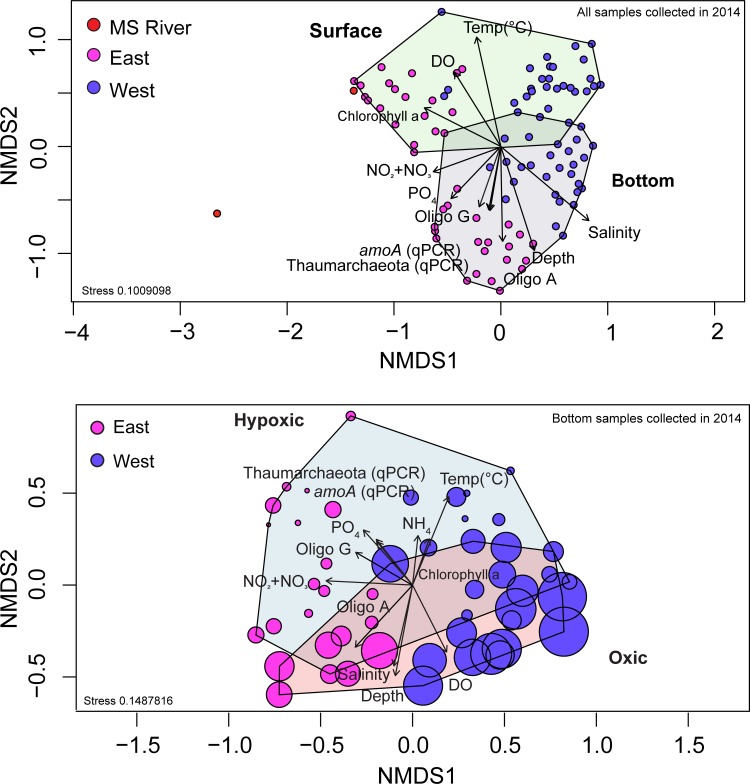
NMDS ordination of normalized 16S rRNA gene iTag sequence data. **(A)** NMDS ordination of normalized 16S rRNA gene iTag sequence data for all Y14 surface and bottom samples. Mississippi River samples (MS) are in red **(A)**, samples east of the Atchafalaya River (AR) are in magenta and samples west of the AR are in purple. **(B)** NMDS ordination of normalized 16S rRNA gene iTag sequence data of Y14 bottom samples only. Bubble sizes are proportional to DO concentration. All environmental variables shown as vectors were significantly (corrected p-values ≤ 0.05) correlated with an NMDS axis for both **(A)** and **(B)**.

To examine the primary drivers in structuring the 2014 microbial communities in the surface and in the bottom water nGOM hypoxic zone, whole community 16S rRNA gene sequence data were examined using Bray-Curtis distances with non-metric multidimensional scaling (NMDS). All environmental variables shown as vectors in Fig 2 were significantly correlated (corrected p-values ≤ 0.05) with NMDS axes. The primary drivers in influencing the microbial community structure were DO, depth, NO_2_+NO_3_, and PO_4_ (Fig 2 and Table H in S2 File), with NO_2_+NO_3_ and PO_4_ decreasing with increasing distance from the MS river mouth. While the adonis test for all Y14 samples was significant, so too was beta-dispersion, suggesting non-homogenous dispersion for these sample clusters, therefore these clusters are not interpreted to be significant.

To analyze beta diversity in Y14 bottom water hypoxic versus oxic conditions, surface samples were excluded and bottom water samples were examined using NMDS ordination (Fig 2B). An adonis test revealed distinct microbial communities in hypoxic samples as compared to oxic water samples (Fig 2B) (adonis R^2^ = 0.09 and p-value ≤ 0.05, beta-dispersion F = 0.60 p-value ≥ 0.05). Bottom samples showed significant spatial clustering east and west of the AR (adonis R^2^ = 0.35 and p-value ≤ 0.05, beta-dispersion F = 0.01 p-value ≥ 0.05) (Fig 2B). We acknowledge that there are many drivers, beyond low oxygen, influencing the community composition, some of which cannot be distilled from ordination. For example, the direct interplay between oxygen, the microbial community, phytoplankton and abiotic variables is not captured in the ordination. However, these ordinations represent the strength of the measured variables correlating with changes in the microbial community.

### Quantitative 16S rRNA and archaeal *amoA* gene abundances in the 2014 hypoxic zone

Bacterial and thaumarchaeal 16S rRNA and archaeal *amoA* gene copy numbers (qPCR) were determined in surface and bottom water samples. Bacterial 16S rRNA gene copy numbers L^-1^ were similar in the surface (avg. 4.10 × 10^7^) and in the bottom water samples (avg. 3.73 × 10^7^). In contrast, thaumarchaeal 16S rRNA gene copy numbers L^-1^ were significantly higher in bottom water samples (avg. 2.18 × 10^7^) compared to surface samples (avg. 2.19 × 10^6^), as were *amoA* copy numbers L^-1^ (bottom avg. 3.12 × 10^7^ and surface avg. 2.96 × 10^6^) (Wilcoxon, Table B in S2 File). When comparing hypoxic and oxic samples in bottom water only, thaumarchaeal 16S rRNA gene copy numbers L^-1^ were significantly higher in hypoxic water samples (avg. 3.28 × 10^7^) compared to oxic water samples (avg. 8.87 × 10^6^) as were *amoA* copy numbers L^-1^ (avg. hypoxic 4.65 × 10^7^ and avg. oxic 1.32 × 10^7^) (Table B in S2 File). In surface and bottom water samples, and in bottom water only samples, thaumarchaeal 16S rRNA and *amoA* gene copy numbers L^-1^ were significantly positively correlated with each other, NO_2_+NO_3_ and PO_4_, and significantly inversely correlated with DO (Tables D-G in S2 File). The ratio of Thaumarchaeota 16S rRNA:*amoA* gene copy number L^-1^ was one (avg.).

### Differences in the extent of hypoxia and microbial community structure between years 2013 and 2014

The total area of low oxygen in Y14 was 13,080 km^2^, compared to 15,120 km^2^ in Y13 (gulfhypoxia.net). Comparing the same hypoxic sites sampled in both Y13 and Y14 revealed that DO in Y13 samples was lower (avg. 0.62mg/L) than Y14 (avg. 1.1mg/L) and the average depth of the hypoxic zone was deeper in Y13 (17.7 mbsl) compared to Y14 (14.6 mbsl) (Figure D in S1 File). Ammonium, salinity and temperature were significantly different between the two hypoxic zones (Wilcoxon, Table B in S2 File) with average NH_4_ concentrations being higher in Y13. In the Y13 hypoxic zone, the average ammonium and salinity concentrations were higher, while in the Y14 hypoxic zone (which was located in shallower waters) the average temperature was higher.

Alpha diversity, Shannon, observed species richness, and equitability were not statistically significantly different between the years. To look at beta diversity between the years, the bottom samples collected at the same stations in Y13 and Y14 were examined using NMDS (Figure E in S1 File), with environmental variables that were significantly correlated with NMDS axes represented as vectors (corrected p-values ≤ 0.05, Table H in S2 File). An adonis test revealed distinct microbial communities in Y14 samples as compared to Y13 samples (Figure EA in S1 File) (adonis R^2^ = 0.20 and p-value ≤ 0.05, beta-dispersion F = 1.07 p-value ≥ 0.05). Whereas Y14 showed distinct east and west clusters, Y13 did not (adonis R^2^ = 0.09 and p-value ≤ 0.05, beta-dispersion F = 10.47 p-value ≤ 0.05) (Figure EA in S1 File).

Analysis of normalized iTag sequence data of bottom samples collected at the same stations in Y13 and Y14 revealed that the phyla Thaumarchaeota, Actinobacteria, Planctomycetes, Euryarchaeota, and SAR406 were more abundant in Y13 than Y14, whereas Cyanobacteria, Proteobacteria, and Bacteroidetes were more abundant in Y14. At the OTU level, 17 had significantly different normalized abundances between Y13 and Y14 (Wilcoxon). Five of the 17 OTUs were more abundant in Y14, whereas 12 OTUs were more abundant in Y13 (Figure F in S1 File). Specifically, the *N*. *maritimus* OTU4369009 (Figure F in S1 File), a Thaumarchaeota OTU4073697 (95% similar to cultured representative *Nitrosopelagicus brevis* strain CN25), and a MGII Euryarchaeota OTU3134564 had higher normalized abundances in Y13 than in Y14. Of the five OTUs that had higher abundances in Y14, one was another Thaumarchaeota OTU1584736 (also 95% similar to cultured representative *Nitrosopelagicus brevis* strain CN25).

When comparing absolute abundance data in the same hypoxic sites sampled in both years, thaumarchaeal 16S rRNA and archaeal *amoA* gene copy numbers (qPCR) per L averages were significantly higher (Figure D in S1 File and Table B in S2 File) in Y14 (avg. 3.31 × 10^7^ and 4.52 × 10^7^) than in Y13 (avg. 7.25 × 10^6^ and 6.87 × 10^6^). In both years, copy number per L of each gene was significantly inversely correlated with DO (Spearman’s ρ and corrected p-values in Tables I-L in S2 File).

### Shannon entropy analysis of Nitrosopumilus 16S rRNA gene sequence data in the 2013 and 2014 hypoxic zones

**Fig 3:**
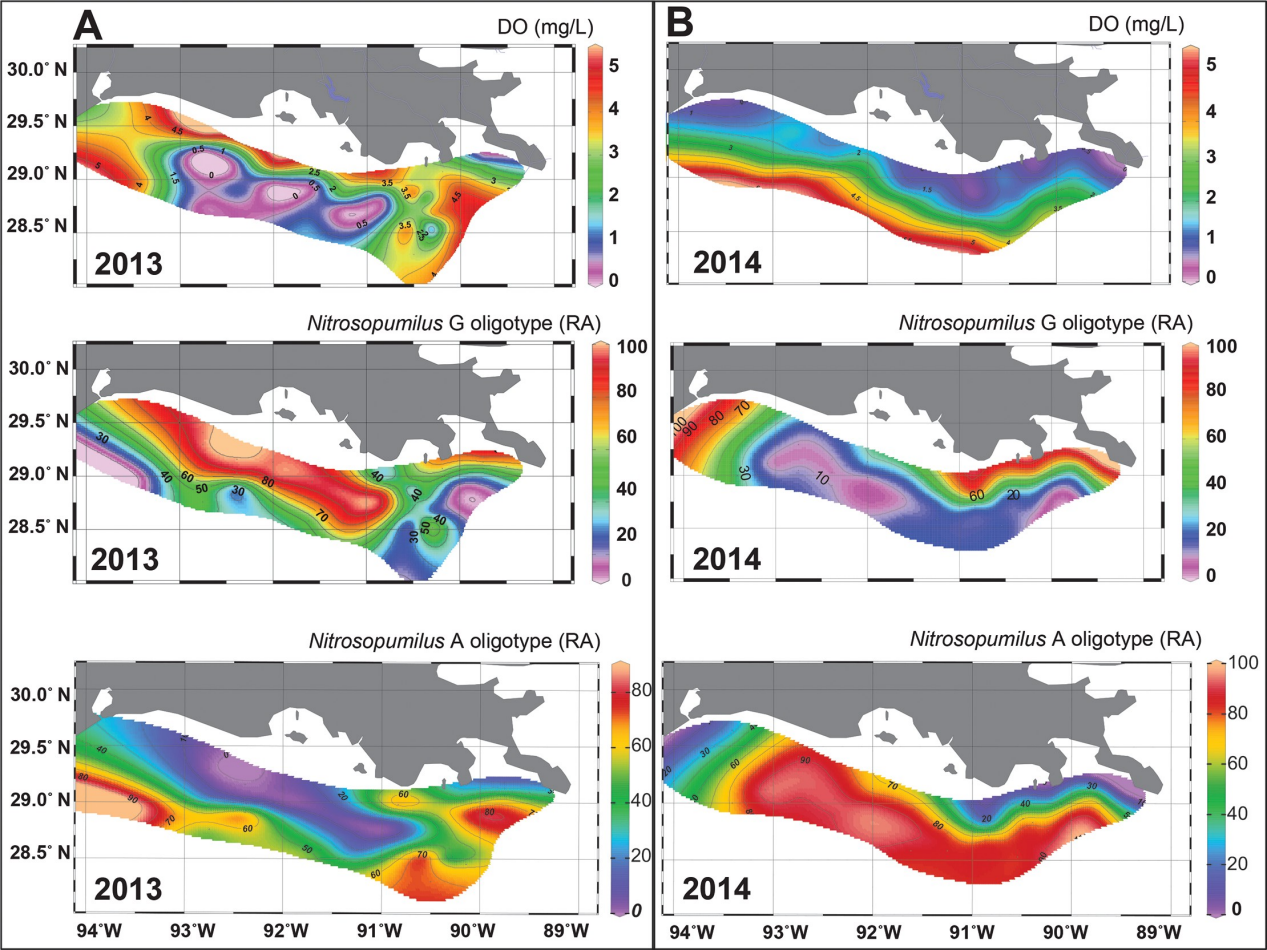
Ocean Data View plots of DO and *Nitrosopumilus* G and A oligotype data. Plots of **(A)** DO concentrations and oligotype data for *Nitrosopumilus* G and A oligotypes for the same samples collected in Y13 **(B)** DO concentrations and oligotype data for *Nitrosopumilus* G and A oligotypes for the same samples collected in Y14.

Due to the high abundances of *Nitrosopumilus* in hypoxic samples in Y13 and Y14, oligotyping analysis was carried out to examine concealed diversity among closely related *Nitrosopumilus* (all sequences annotated as such) in relationship to environmental variables by analyzing subtle nucleotide variations among 16S rRNA gene sequences. The Y13 iTag data were not analyzed in this way in our previous paper [19], so here we present CSS normalized OTU data oligotyping results for both Y13 and Y14 in the same station samples (n = 35/year). The normalized abundance of oligotype G (G in nt position 115/250) was significantly higher in abundance in hypoxic samples and more abundant in Y13 compared to Y14 (Wilcoxon, Table B in S2 File). Oligotype G was significantly inversely correlated with DO in both Y13 and Y14 (Tables I-L in S2 File). Conversely, oligotype A (A in nt position 115/250) was lower in hypoxic samples in both years (Fig 3). Oligotypes T or C at nt position 115/250 were lower, reaching maximal abundances of 6.45% and 0.29%, respectively. The oligotyping analysis was then extended to include all *Nitrosopumilaceae*, which include the two Thaumarchaeota OTUs that were significantly different between years and 95% similar to *Nitrosopelagicus brevis* (OTU4073697 and OTU1584736). This extended analysis identified two abundant oligotypes, G and A, neither of which were significantly different between hypoxic and oxic samples, nor significantly correlated with DO (normalized abundances). However, these oligotypes G and A were significantly different between Y13 and Y14, with oligotype G being more abundant in Y13 and oligotype A being more abundant in Y14 (Figure G in S1 File).

### Thaumarchaeota AOA microbial species co-occurrence patterns in the 2013 and 2014 hypoxic zones

**Fig 4:**
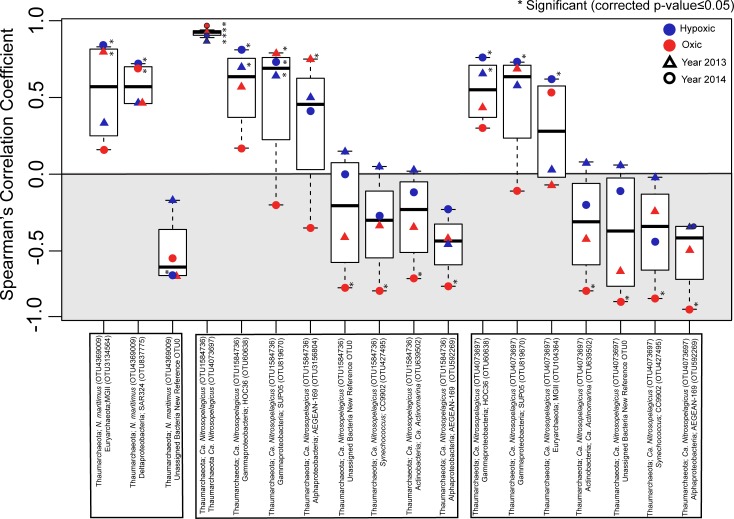
Plots representing co-occurrence (Spearman’s ρ) data for OTUs of interest. Co-occurrence (Spearman’s ρ) of Thaumarchaeota (*N*. *maritimus* OTU4369009, *Ca*. *Nitrosopelagicus* OTU1584736, and *Ca*. *Nitrosopelagicus* OTU4073697) and ten OTUs whose normalized abundances were significantly different between years (Wilcoxon test with B-H corrected p-values) in the same Y13 and Y14 bottom water samples. Correlations that were significant (corrected p-values ≤ 0.05) are indicated by an asterisk.

We evaluated species co-occurrences by determining Spearman’s correlation coefficients for the same Y13 and Y14 bottom water sites (n = 35/year). Similar to oligotyping analysis, species co-occurrence was not previously considered in our Y13 dataset. For this analysis, we included only the 17 OTUs discussed above that were significantly different between the years (Wilcoxon), which included the three Thaumarchaeota OTUs; 4369009, 1584736, 4073697 (Figure F in S1 File) to understand how changes in normalized abundance of these microorganisms between two different hypoxic seasons could be related to co-occurrences with other significantly different microbial OTUs between the two years (Fig 4). Of the 17 OTUs that were significantly different between Y13 and Y14 same station samples, ten OTUs were significantly correlated at least once with one of the three Thaumarchaeota OTUs in Y13 and/or Y14 hypoxic or oxic samples (Fig 4).

## Discussion

The Y14 hypoxic zone was slightly smaller in size (13,080 km^2^), closer to shore, and discontinuous as compared to the Y13 hypoxic zone (15,120 km^2^) (Figure D in S1 File). In July 2014, wind speeds reached between 10 to 20 knots blowing from the west, with higher than average wave height (1.4 meters) reported (http://www.wavcis.lsu.edu/). Further, in Y14 there was an unseasonably late (late July), above average Mississippi River discharge (avg. is ~ 4 x 10^5^ ft^2^/sec compared to 5 x 10^5^ ft^2^/sec in Y14), which resulted in nitrogen (NO_2_ + NO_3_) concentrations reaching a near-record high (200 μmol L^-1^) and an anomalously high phytoplankton biomass (e.g., 118 ug/L Chl *a* at the end of July) (LUMCON 2014, http://bit.ly/2wHLSk1, accessed 02/08/17) late in the hypoxic season (Figure A in S1 File and Table A in S2 File). These factors could have resulted in the slight DO replenishment seen between 92° W and 92.38° W, specifically along transect G, west of the Atchafalaya River (AR) (Figure A in S1 File and Table A in S2 File). Previous reports of wind out of the west and southwest during the summer months have correlated with a smaller hypoxic zone, which moves nutrient enhanced water masses to the east and to deeper waters, disrupting density stratification [71,72]. Therefore, a plausible hypothesis is that the variability in wind forcing resulted in the movement of water masses to the east [73]. This wind forcing could have influenced the hypoxic area to the west of the Atchafalaya River, resulting in the variability in the size and shape of the hypoxic zone between Y13 and Y14, while also potentially shaping the microbial community as seen in the east west latitude clustering in Y14, but not in Y13 (Figures D and E in S1 File).

Specifically, our data revealed a significant inverse correlation between AOA and DO in the nGOM. However, in Y14 when the hypoxic zone was less extensive and focused at the MS River mouth, the normalized abundances of AOA were lower than in Y13. In Y14, although AOA were enriched in the hypoxic bottom water, after correcting for multiple comparisons, their normalized abundances (iTag) were not significantly higher in hypoxic versus oxic samples. Yet the absolute abundances of thaumarchaeal 16S rRNA and *amoA* genes were significantly higher in Y14 than in Y13 (Figure D in S1 File). It is also important to recognize that the qPCR data quantifies absolute abundances, compared to the normalized 16S rRNA gene data, which only quantifies relative abundances. These two different types of data may have led to the discrepancy between iTag and qPCR datasets. Another theory for the difference between iTag and qPCR data could be that the iTag primers did not discern the full breadth of Thaumarchaeota diversity in comparison to archaeal qPCR primers.

Further comparison of Y13 and Y14 revealed a single nucleotide variation among all *Nitrosopumilus* 16S rRNA gene sequences. Oligotyping analysis demonstrated *Nitrosopumilus* oligotype G to be both annually abundant in the nGOM hypoxic zone and significantly inversely correlated with DO (Table B in S2 File), whereas oligotype A was not (Fig 3). These oligotypes were differentiated by 1 bp in all 16S rRNA gene sequences annotated as *Nitrosopumilus*, suggesting previously unrecognized genomic diversity between closely related *Nitrosopumilus* across environmental gradients in the nGOM. The data suggested that oligotype G abundance was influenced by the severity of bottom water hypoxia (expansive and deeper versus shallow and smaller), with higher abundances in Y13 (Fig 3). Therefore, subtle nucleotide variations, such as that presented herein could be indicative of a ubiquitous and diverse *Nitrosopumilus* population where *Nitrosopumilus* oligotype (G) is adapted to low DO. Although the other *Nitrosopumilaceae* oligotypes identified here were not significantly correlated with oxygen or other measured environmental variables, their normalized abundances did differ significantly by year, further supporting subtle AOA genetic diversity where subpopulations of *Nitrosopumilaceae* fluctuate in their abundances between hypoxic seasons (Figure G in S1 File). However, the environmental drivers of the distribution of the *Nitrosopumilaceae* oligotypes remain to be determined. Previously, oligotyping has revealed ecologically important sub-OTUs in human and environmental microbiomes across environmental gradients [68,74–77]. For example, Sintes and colleagues [76] identified two groups of *amoA* oligotypes that clustered according to high or low latitude and subclustered by ocean depth, however little other oligotypic analysis has been carried out on AOA, specifically *Nitrosopumilus*. Whether the low DO prevalent AOA oligotype that was dominant in coastal hypoxic samples is ecologically consequential remains to be determined.

Microbial species co-occurrences relationships can reveal community patterns, and facilitate hypothesis generation regarding abiotic influences on random and non-random patterns, as well as microbial interactions [63–66]. In our study, co-occurrence analysis of OTUs that were significantly different between bottom water sites in Y13 and Y14 revealed that increasing normalized abundances of *N*. *maritimus* OTU4369009, particularly in Y13, were closely matched with changes in abundance of other OTUs, specifically MGII Euryarchaeota OTU3134564 and Deltaproteobacteria OTU837775 (putatively identified as a SAR324 by SILVA), suggesting changes in abundances of these OTUs may influence one another (Fig 4). Previous studies reported that MGII have an aerobic photoheterotrophic lifestyle [78–80] with higher abundances in the euphotic zone compared to depths below the euphotic zone [25,81–83]. Whereas Thaumarchaeota, closely related to the *N*. *maritimus* are known to increase in abundance with depth in the open ocean [16,17,25,81,84]. In contrast to these findings we see high thaumarchaeal abundances in our shallow (avg. depth 19 mbsl), euphotic zone water column samples, therefore our findings of higher MGII abundances in the euphotic zone are consistent with reports of MGII abundances. However, in our Y14 samples MGII were not as abundant as in Y13, yet Thaumarchaeota were abundant in both years. If there is any metabolic exchange between these two marine microbes, the low abundance of MGII in a given year could potentially lead to metabolic linkages being decoupled, which could be ecologically significant. Also, It has been reported that some SAR324 have the ability to oxidize hydrogen sulfide [85] which could, in theory, mean that when hypoxic conditions prevail and AOA continues to draw down DO, SAR324 could oxidize sulfide that may flux from the sediments resulting in detoxification of bottom water. During hypoxic conditions when *N*. *maritimus* OTU4369009 abundances are high, co-occurrence with SAR324 abundances are weakened (Fig 4) and sulfide oxidation by SAR324 may not keep pace, resulting in a deteriorating environment beyond low DO.

The abundance of two Thaumarchaeota (OTU1584736 and OTU4073697) were significantly different between the years (Figure F in S1 File); however they were not significantly different between hypoxic and oxic samples nor were they correlated with DO. In both years they were positively correlated with NO_2_+NO_3_, and in Y14 they were positively correlated with PO_4_ and inversely correlated with NH_4_ (Tables I-L in S2 File). Therefore, variations in nutrient type and concentration between hypoxic seasons in the nGOM could be a driver in shaping these two Thaumarchaeota OTU abundances between years. Higher abundances in relationship to nutrient concentrations in certain years could be ecologically consequential given that close relatives have been shown to produce N_2_O. [39]. For example, these two OTUs are 95% similar to the *N*. *Brevis* CN25 [86], an AOA that has been shown to produce N_2_O in enrichment cultures [39], similar to *N*. *maritimus* [40,87]. In fact, AOA have been reported to be the primary source of N_2_O in the surface ocean [39] and it has been shown that decreasing DO could influence the production of N_2_O by AOA [30,39,40,88,89]. In the shallow nGOM water column, Walker and colleagues [90] reported that nitrification was the primary source of N_2_O during peak hurricane season, consistent with the results of Pakulski (2000) [22]. Therefore, these two Thaumarchaeota OTUs as well as the Thaumarchaeota OTU4369009 discussed previously, that we show to be abundant in the nGOM hypoxic zone of both years may contribute to N_2_O production, a potent greenhouse gas [91,92], which can flux from ocean to the atmosphere when the water column is mixed, such as during hurricanes or tropical storm activity which occur around the same time as hypoxia formation [90].

Additionally, a decoupling between the two-step transformation of ammonium to nitrate (which is carried out by distinct groups of microorganisms) has previously been reported (e.g. [93], including in the nGOM hypoxic zone [18]. In our dataset, nitrite oxidizing bacteria (NOB) such as *Nitrospina* had low relative abundances (< 2%), and the comammox bacterium *Nitrospira*, were undetectable. Thus, the annual increase in AOA in the nGOM, and the lack of co-occurrence with NOB, is consistent with a general microbial community pattern that leads to nitrite accumulation, as shown by Bristow and colleagues [18] in this expansive hypoxic zone.

## Conclusion

Collectively, this dataset supports the conclusion that the nGOM hypoxic zone can serve as a hotspot for AOA. Furthermore, the normalized abundance of Thaumarchaeota 16S rRNA (iTag) and the absolute abundance (qPCR) of Thaumarchaeota 16S rRNA and archaeal *amoA* gene copy numbers can reflect the extent of bottom water hypoxia. This study adds to the knowledge on Thaumarchaeota populations in coastal environments, such as in polar environments [94–98] and the in US waters of Puget Sound in WA [99] and coastal GA [100–102]. Our findings of an increase in Thaumarchaeota in the hypoxic nGOM are consistent with several studies that have reported an increase in abundance of AOA in low oxygen marine environments [17–19,32–35,38,103], as well as with cultivation based analyses that have shown Thaumarchaeota growth at low O_2_ concentrations [30]. While there is precedence for Thaumarchaeota thriving in low oxygen environments, this is the first report where high abundances of AOA were determined in a shallow (avg. depth 19 mbsl), sub-tropical hypoxic zone over two consecutive years. In a changing climate that leads to exponentially expanding OMZs [1,2,104–106], future studies are needed to determine the ecological implications of an AOA hotspot in these OMZs, including how high AOA abundances perturb co-occurrence relationships with other microbial groups. Given that N_2_O impacts climate and could lead to a positive feedback loop [1,2,104–106] quantifying AOA N_2_O production in a shallow OMZ, such as in the nGOM, is critical.

## Supporting information

S1 File Supplementary figures. **Figure A. (A)** Sample map of the 52 stations sampled during the Y14 nGOM shelfwide cruise, in which bottom water status is indicated (red circles indicates oxic stations while blue circles indicates hypoxic stations). **(B)** DO concentrations from the bottom samples collected. **Figure B. (A)** NMDS ordination of normalized 16S rRNA gene iTag sequence data for all samples collected in year 2014 grouped by surface and bottom. The seventeen bubble plots represent the same NMDS plot with normalized abundances of the OTUs that were statistically significantly different (Wilcoxon) between surface and bottom samples depicted by bubble size, where larger bubble size represents higher normalized abundances. The symbol * represents OTUs that were statistically significantly inversely correlated with dissolved oxygen (Spearman correlation; B-H corrected p-value ≤ 0.05). **Figure C. (A)** NMDS ordination of normalized 16S rRNA gene iTag sequence data for the all bottom samples collected in year 2014 where bubble size represents DO concentration. The other sixteen NMDS bubble plots represent the normalized abundances of the OTUs that were statistically significantly different (Wilcoxon) between hypoxic and oxic samples. Larger bubble size represents higher normalized abundances. The symbol * represents OTUs that were statistically significantly inversely correlated with dissolved oxygen while the other OTUs are significantly positively correlated with DO (Spearman correlation; B-H corrected p-value ≤ 0.05). **Figure D.** Plots of DO concentrations, relative abundances of Nitrosopumilus OTU4369009 16S rRNA genes (iTag), Thaumarchaeota 16S rRNA and amoA gene copy number/L (qPCR) for bottom water samples collected at the same sites in Y13 and Y14. **Figure E. (A)** NMDS ordination of normalized 16S rRNA gene iTag sequence data for the same samples collected in Y13 and Y14 with environmental variables, qPCR data and oligotype data shown as vectors. All environmental variables represented as vectors on the NMDS were significantly correlated (corrected p-value ≤ 0.05) with an NMDS axis. (B) The same NMDS ordination depicting oxygen concentration (DO) as bubble size e.g. larger bubbles indicate higher DO concentrations. **Figure F. (A)** NMDS ordination of normalized 16S rRNA gene iTag sequence data for the same samples collected in Y13 and Y14 where bubble size depicts oxygen concentration. The other seventeen NMDS bubble plots represent the normalized abundances of the OTUs that were statistically significantly different (Wilcoxon) between the years where larger bubble size represents higher normalized abundances. The symbol * represents OTUs that were statistically significantly inversely correlated with DO in Y14, and the symbol # represents a significant inverse correlation with DO in Y13 (Spearman correlation; B-H corrected p-value ≤ 0.05). **Figure G.** Plots of **(A)** Oligotype data for *Nitrosopumilaceae* G and A oligotypes for the same samples collected in Y13 **(B)** Oligotype data for *Nitrosopumilaceae* G and A oligotypes for the same samples collected in Y14.
(PDF)

S2 File Supplementary tables. **Table A**. 2014 nGOM hypoxic zone sample metadata and in situ chemistry. **Table B**. Wilcoxon B-H corrected p-values for diversity statistics and environmental variables for all datasets. **Table C**. Operational taxonomic unit (OTU) table for all samples collected in 2014. **Table D**. P-values (B-H corrected) for Spearman's correlation coefficients for Y14 surface and bottom samples. **Table E**. Spearman's Correlation coefficients (ρ) for Y14 surface and bottom samples. **Table F**. P-values (B-H corrected) for Spearman's correlation coefficients for Y14 bottom samples only. **Table G**. Spearman's Correlation coefficients (ρ) for Y14 bottom samples only. **Table H**. Envfit B-H corrected p-values for NMDS ordinations. **Table I**. P-values (B-H corrected) for Spearman's correlation coefficients for Y13 same samples only. **Table J**. Spearman's Correlation coefficients (ρ) for Y13 same samples only. **Table K**. P-values (B-H corrected) for Spearman's correlation coefficients for Y14 same samples only. **Table L**. Spearman's Correlation coefficients (ρ) for Y14 same samples only.
(XLSX)

## References

[1] Levin LA, Breitburg DL. Linking coasts and seas to address ocean deoxygenation. Nat Clim Chang. 2015;5(5):401–3.

[2] Breitburg D, Levin LA, Oschlies A, Grégoire M, Chavez FP, Conley DJ, et al Declining oxygen in the global ocean and coastal waters. Science. 2018;359(6371):eaam7240 10.1126/science.aam7240 29301986

[3] Rabalais NN, Turner RE, Scavia D. Beyond Science into Policy: Gulf of Mexico Hypoxia and the Mississippi River. Bioscience. 2002;52(2):129.

[4] Turner RE, Rabalais NN, Justic D. Predicting summer hypoxia in the northern Gulf of Mexico: Riverine N, P, and Si loading. Mar Pollut Bull. 2006;52(2):139–48. 10.1016/j.marpolbul.2005.08.012 16212987

[5] Wright JJ, Konwar KM, Hallam SJ. Microbial ecology of expanding oxygen minimum zones. Nat Rev Microbiol. 2012;10(6):381–94. 10.1038/nrmicro2778 22580367

[6] Diaz RJ, Rosenberg R. Spreading dead zones and consequences for marine ecosystems. Science. 2008;321(5891):926–9. 10.1126/science.1156401 18703733

[7] Rabalais NN, Turner RE, Wiseman WJ. Gulf of Mexico Hypoxia, A.K.A. “The Dead Zone.” Annu Rev Ecol Syst. 2002;33(1):235–63.

[8] Rabalais NN, Turner RE, Wiseman WJ. Hypoxia in the Gulf of Mexico. J Environ Qual. 2001;30(2):320 11285891 10.2134/jeq2001.302320x

[9] Rabalais NN, Turner RE. Oxygen depletion in the Gulf of Mexico adjacent to the Mississippi River In: Neretin LN, editor. Past and present water column anoxia. Springer Netherlands; 2006 p. 225–45.

[10] Dagg M, Sato R, Liu H, Bianchi TS, Green R, Powell R. Microbial food web contributions to bottom water hypoxia in the northern Gulf of Mexico. Cont Shelf Res. 2008;28(9):1127–37.

[11] Dagg MJ, Ammerman JW, Amon RMW, Gardner WS, Green RE, Lohrenz SE. A review of water column processes influencing hypoxia in the northern Gulf of Mexico. Estuaries and Coasts. 2007 10;30(5):735–52.

[12] Rabalais NN, Turner RE, Wiseman William J J. Characterization and long-term trends of hypoxia in the northern Gulf of Mexico: Does the science support the Action Plan? Estuaries and Coasts. 2007;30(5):753–72.

[13] Chen N, Bianchi TS, McKee BA, Bland JM. Historical trends of hypoxia on the Louisiana shelf: application of pigments as biomarkers. Org Geochem. 2001;32(4):543–61.

[14] Rabalais NN, Turner RE, Dortch Q, Justic D, Bierman VJ, Wiseman WJ. Nutrient-enhanced productivity in the northern Gulf of Mexico: past, present and future. In: Nutrients and Eutrophication in Estuaries and Coastal Waters. Dordrecht: Springer Netherlands; 2002 p. 39–63.

[15] Wiseman WJ, Rabalais NN, Turner RE, Dinnel SP, MacNaughton A. Seasonal and interannual variability within the Louisiana coastal current: stratification and hypoxia. J Mar Syst. 1997;12(1–4):237–48.

[16] King GM, Smith CB, Tolar B, Hollibaugh JT. Analysis of Composition and Structure of Coastal to Mesopelagic Bacterioplankton Communities in the Northern Gulf of Mexico. Front Microbiol. 2013;3:438 10.3389/fmicb.2012.00438 23346078 PMC3548560

[17] Tolar BB, King GM, Hollibaugh JT. An analysis of thaumarchaeota populations from the northern gulf of Mexico. Front Microbiol. 2013;4:72 10.3389/fmicb.2013.00072 23577005 PMC3620491

[18] Bristow LA, Sarode N, Cartee J, Caro-Quintero A, Thamdrup B, Stewart FJ. Biogeochemical and metagenomic analysis of nitrite accumulation in the Gulf of Mexico hypoxic zone. Limnol Oceanogr. 2015;60(5):1733–50.

[19] Gillies LE, Thrash JC, de Rada S, Rabalais NN, Mason OU. Archaeal enrichment in the hypoxic zone in the northern Gulf of Mexico. Environ Microbiol. 2015; 17(10), 3847–3856. 10.1111/1462-2920.12853 25818237

[20] Thrash JC, Seitz KW, Baker BJ, Temperton B, Gillies LE, Rabalais NN, et al Metabolic Roles of Uncultivated Bacterioplankton Lineages in the Northern Gulf of Mexico "Dead Zone";. MBio. 2017;8(5):e01017–17. 10.1128/mBio.01017-17 28900024 PMC5596340

[21] Könneke M, Bernhard AE, de la Torre JR, Walker CB, Waterbury JB, Stahl D a. Isolation of an autotrophic ammonia-oxidizing marine archaeon. Nature. 2005;437(7058):543–6. 10.1038/nature03911 16177789

[22] Pakulski JD, Benner R, Whitledge T, Amon R, Eadie B, Cifuentes L, et al Microbial Metabolism and Nutrient Cycling in the Mississippi and Atchafalaya River Plumes. Estuar Coast Shelf Sci. 2000;50(2):173–84.

[23] Nunnally CC, Quigg A, DiMarco S, Chapman P, Rowe GT. Benthic–pelagic coupling in the Gulf of Mexico hypoxic area: Sedimentary enhancement of hypoxic conditions and near bottom primary production. Cont Shelf Res. 2014;85:143–52.

[24] Francis CA, Roberts KJ, Beman JM, Santoro AE, Oakley BB. Ubiquity and diversity of ammonia-oxidizing archaea in water columns and sediments of the ocean. Proc Natl Acad Sci. 2005;102(41):14683–8. 10.1073/pnas.0506625102 16186488 PMC1253578

[25] Karner MB, DeLong EF, Karl DM. Archaeal dominance in the mesopelagic zone of the Pacific Ocean. Nature. 2001;409(6819):507–10. 10.1038/35054051 11206545

[26] Wuchter C, Abbas B, Coolen MJL, Herfort L, van Bleijswijk J, Timmers P, et al Archaeal nitrification in the ocean. Proc Natl Acad Sci U S A. 2006 8;103(33):12317–22. 10.1073/pnas.0600756103 16894176 PMC1533803

[27] Mincer TJ, Church MJ, Taylor LT, Preston C, Karl DM, DeLong EF. Quantitative distribution of presumptive archaeal and bacterial nitrifiers in Monterey Bay and the North Pacific Subtropical Gyre. Environ Microbiol. 2007;9(5):1162–75. 10.1111/j.1462-2920.2007.01239.x 17472632

[28] Church MJ, Wai B, Karl DM, DeLong EF. Abundances of crenarchaeal *amoA* genes and transcripts in the Pacific Ocean. Environ Microbiol. 2010;12(3):679–88. 10.1111/j.1462-2920.2009.02108.x 20002133 PMC2847202

[29] Martens-Habbena W, Berube PM, Urakawa H, de la Torre JR, Stahl D a. Ammonia oxidation kinetics determine niche separation of nitrifying Archaea and Bacteria. Nature. 2009;461(7266):976–9. 10.1038/nature08465 19794413

[30] Qin W, Meinhardt KA, Moffett JW, Devol AH, Virginia Armbrust E, Ingalls AE, et al Influence of oxygen availability on the activities of ammonia-oxidizing archaea. Environ Microbiol Rep. 2017;9(3):250–6. 10.1111/1758-2229.12525 28211189

[31] Qin W, Carlson LT, Armbrust EV, Devol AH, Moffett JW, Stahl DA, et al Confounding effects of oxygen and temperature on the TEX_86_ signature of marine Thaumarchaeota. Proc Natl Acad Sci U S A. 2015;112(35):10979–10984. 10.1073/pnas.1501568112 26283385 PMC4568219

[32] Bristow LA, Dalsgaard T, Tiano L, Mills DB, Bertagnolli AD, Wright JJ, et al Ammonium and nitrite oxidation at nanomolar oxygen concentrations in oxygen minimum zone waters. Proc Natl Acad Sci U S A. 2016;113(38):10601–6. 10.1073/pnas.1600359113 27601665 PMC5035861

[33] Belmar L, Molina V, Ulloa O. Abundance and phylogenetic identity of archaeoplankton in the permanent oxygen minimum zone of the eastern tropical South Pacific. FEMS Microbiol Ecol. 2011 11;78(2):314–26. 10.1111/j.1574-6941.2011.01159.x 21696407

[34] Molina V, Belmar L, Ulloa O. High diversity of ammonia-oxidizing archaea in permanent and seasonal oxygen-deficient waters of the eastern South Pacific. Environ Microbiol. 2010;12(9):2450–65. 10.1111/j.1462-2920.2010.02218.x 20406296

[35] Lam P, Jensen MM, Lavik G, McGinnis DF, Müller B, Schubert CJ, et al Linking crenarchaeal and bacterial nitrification to anammox in the Black Sea. Proc Natl Acad Sci U S A. 2007;104(17):7104–9. 10.1073/pnas.0611081104 17420469 PMC1849958

[36] Lam P, Lavik G, Jensen MM, van de Vossenberg J, Schmid M, Woebken D, et al Revising the nitrogen cycle in the Peruvian oxygen minimum zone. Proc Natl Acad Sci U S A. 2009;106(12):4752–7. 10.1073/pnas.0812444106 19255441 PMC2649953

[37] Stewart FJ, Ulloa O, DeLong EF. Microbial metatranscriptomics in a permanent marine oxygen minimum zone. Environ Microbiol. 2012;14(1):23–40. 10.1111/j.1462-2920.2010.02400.x 21210935

[38] Beman JM, Popp BN, Francis C a. Molecular and biogeochemical evidence for ammonia oxidation by marine Crenarchaeota in the Gulf of California. ISME J. 2008;2(4):429–41. 10.1038/ismej.2007.118 18200070

[39] Santoro AE, Buchwald C, McIlvin MR, Casciotti KL. Isotopic Signature of N2O Produced by Marine Ammonia-Oxidizing Archaea. Science. 2011;333(6047). 10.1126/science.1208239 21798895

[40] Löscher CR, Kock A, Könneke M, Laroche J, Bange HW, Schmitz RA. Production of oceanic nitrous oxide by ammonia-oxidizing archaea. Biogeosciences. 2012;9:2419–29.

[41] Parsons TR, Maita Y, Lalli CM. A manual of chemical and biological methods for seawater analysis. Pergamon Press. 1984; 173 p.

[42] Arar EJ, Collins GB. In vitro determination of chlorophyll a and pheophytin a in marine and freshwater algae by fluorescence. US Environ Prot Agency Method 4450 Revis 12 1997;1–22.

[43] Schlitzer R. Ocean Data View. http://odv.awi.de. 2013.

[44] Caporaso JG, Lauber CL, Walters WA, Berg-Lyons D, Lozupone CA, Turnbaugh PJ, et al Global patterns of 16S rRNA diversity at a depth of millions of sequences per sample. Proc Natl Acad Sci U S A. 2011;108 Suppl:4516–22. 10.1073/pnas.1000080107 20534432 PMC3063599

[45] Caporaso JG, Lauber CL, Walters WA, Berg-Lyons D, Huntley J, Fierer N, et al Ultra-high-throughput microbial community analysis on the Illumina HiSeq and MiSeq platforms. ISME J. 2012;6(8):1621–4. 10.1038/ismej.2012.8 22402401 PMC3400413

[46] Aronesty Erik. Command-line tools for processing biological sequencing data. github. 2011 p. https://github.com/ExpressionAnalysis/ea-utils.

[47] Caporaso JG, Bittinger K, Bushman FD, DeSantis TZ, Andersen GL, Knight R. PyNAST: a flexible tool for aligning sequences to a template alignment. Bioinformatics. 2010;26(2):266–7. 10.1093/bioinformatics/btp636 19914921 PMC2804299

[48] Krohn A. akutils-v1.2: akutils-v1.2: Facilitating analyses of microbial communities through QIIME. 2016.

[49] Rognes T, Flouri T, Nichols B, Quince C, Mahé F. VSEARCH: a versatile open source tool for metagenomics. PeerJ. 2016;4:e2584 10.7717/peerj.2584 27781170 PMC5075697

[50] Mercier C., Boyer F., Bonin A., & Coissac E. SUMATRA and SUMACLUST: fast and exact comparison and clustering of sequences. 2013;27–9.

[51] Kopylova E, Noe L, Touzet H. SortMeRNA: fast and accurate filtering of ribosomal RNAs in metatranscriptomic data. Bioinformatics. 2012;28(24):3211–7. 10.1093/bioinformatics/bts611 23071270

[52] McDonald D, Price MN, Goodrich J, Nawrocki EP, DeSantis TZ, Probst A, et al An improved Greengenes taxonomy with explicit ranks for ecological and evolutionary analyses of bacteria and archaea. ISME J. 2012;6(3):610–8. 10.1038/ismej.2011.139 22134646 PMC3280142

[53] Paulson JN, Stine OC, Bravo HC, Pop M. Differential abundance analysis for microbial marker-gene surveys. Nat Meth. 2013;10(12):1200–2. 10.1038/nmeth.2658 PMC4010126 24076764

[54] Quast C, Pruesse E, Yilmaz P, Gerken J, Schweer T, Yarza P, et al The SILVA ribosomal RNA gene database project: improved data processing and web-based tools. Nucleic Acids Res. 2012;41(D1):D590–6. 23193283 10.1093/nar/gks1219 PMC3531112

[55] Altschul SF, Gish W, Miller W, Myers EW, Lipman DJ. Basic local alignment search tool. J Mol Biol. 1990;215(3):403–10. 10.1016/S0022-2836(05)80360-2 2231712

[56] Shannon CE, Weaver W. The mathemetaical theory of communication 1964 Urbana, IL: The University of Illinois Press, 1–117.

[57] Benjamini Y, Hochberg Y. Controlling the False Discovery Rate: A Practical and Powerful Approach to Multiple Testing. Source J R Stat Soc Ser B. 1995;57(1):289–300.

[58] Arndt D, Xia J, Liu Y, Zhou Y, Guo AC, Cruz J a, et al METAGENassist: a comprehensive web server for comparative metagenomics. Nucleic Acids Res. 2012;W88–95. 10.1093/nar/gks497 22645318 PMC3394294

[59] Hackstadt AJ, Hess AM. Filtering for increased power for microarray data analysis. BMC Bioinformatics. 2009;10(1):11. 19133141 10.1186/1471-2105-10-11 PMC2661050

[60] Oksanen J, Blanchet FG, Friendly M, Kindt R, Legendre P, McGlinn D, Minchin PR, O’Hara RB, Simpson GL, Solymos P SMF. Vegan: Community Ecology Package. R package version. 2017.

[61] Anderson MJ. A new method for non-parametric multivariate analysis of variance. Austral Ecol. 2001;26(1):32–46.

[62] Revelle W. Using the psych package to generate and test structural models. 2018.

[63] Barberán A, Bates ST, Casamayor EO, Fierer N. Using network analysis to explore co-occurrence patterns in soil microbial communities. ISME J. 2012;6(2):343–51. 10.1038/ismej.2011.119 21900968 PMC3260507

[64] Kittelmann S, Seedorf H, Walters WA, Clemente JC, Knight R, Gordon JI, et al Simultaneous amplicon sequencing to explore co-occurrence patterns of bacterial, archaeal and eukaryotic microorganisms in rumen microbial communities. PLoS One. 2013;8(2):e47879 10.1371/journal.pone.0047879 23408926 PMC3568148

[65] Williams RJ, Howe A, Hofmockel KS. Demonstrating microbial co-occurrence pattern analyses within and between ecosystems. Front Microbiol. 2014;5:358 10.3389/fmicb.2014.00358 25101065 PMC4102878

[66] Berry D, Widder S. Deciphering microbial interactions and detecting keystone species with co-occurrence networks. Front Microbiol. 2014;5:219 10.3389/fmicb.2014.00219 24904535 PMC4033041

[67] Eren a M, Maignien L, Sul WJ, Murphy LG, Grim SL, Morrison HG, et al Oligotyping: Differentiating between closely related microbial taxa using 16S rRNA gene data. Methods Ecol Evol. 2013;4(12):1111–9. 10.1111/2041-210X.12114 PMC3864673 24358444

[68] Eren a M, Borisy GG, Huse SM, Mark Welch JL. Oligotyping analysis of the human oral microbiome. Proc Natl Acad Sci. 2014;111(28):E2875–E2884. 10.1073/pnas.1409644111 24965363 PMC4104879

[69] Meadow J. Convert QIIME files into Oligotyping format [Internet]. 2014. Available from: https://github.com/jfmeadow/q2oligo

[70] Suzuki MT, Taylor LT, DeLong EF. Quantitative analysis of small-subunit rRNA genes in mixed microbial populations via 5’-nuclease assays. Appl Environ Microbiol. 2000;66(11):4605–14. 11055900 10.1128/aem.66.11.4605-4614.2000 PMC92356

[71] Feng Y, DiMarco SF, Jackson GA. Relative role of wind forcing and riverine nutrient input on the extent of hypoxia in the northern Gulf of Mexico. Geophys Res Lett. 2012;39(9).

[72] Zhang X, Hetland RD, Marta-Almeida M, DiMarco SF. A numerical investigation of the Mississippi and Atchafalaya freshwater transport, filling and flushing times on the Texas-Louisiana Shelf. J Geophys Res Ocean. 2012;117(C11).

[73] Feng Y, Fennel K, Jackson GA, DiMarco SF, Hetland RD. A model study of the response of hypoxia to upwelling-favorable wind on the northern Gulf of Mexico shelf. J Mar Syst. 2014;131:63–73.

[74] Reveillaud J, Maignien L, Eren MA, Huber JA, Apprill A, Sogin ML, et al Host-specificity among abundant and rare taxa in the sponge microbiome. ISME J. 2014;8(6):1198–209. 10.1038/ismej.2013.227 24401862 PMC4030224

[75] Kleindienst S, Grim S, Sogin M, Bracco A, Crespo-Medina M, Joye SB. Diverse, rare microbial taxa responded to the Deepwater Horizon deep-sea hydrocarbon plume. ISME J. 2016 2;10(2):400–15. 10.1038/ismej.2015.121 26230048 PMC4737931

[76] Sintes E, De Corte D, Haberleitner E, Herndl GJ. Geographic Distribution of Archaeal Ammonia Oxidizing Ecotypes in the Atlantic Ocean. Front Microbiol. 2016;7:77 10.3389/fmicb.2016.00077 26903961 PMC4746290

[77] Schmidt VT, Reveillaud J, Zettler E, Mincer TJ, Murphy L, Amaral-Zettler LA. Oligotyping reveals community level habitat selection within the genus Vibrio. Front Microbiol. 2014;5:563 10.3389/fmicb.2014.00563 25431569 PMC4230168

[78] Frigaard N-U, Martinez A, Mincer TJ, DeLong EF. Proteorhodopsin lateral gene transfer between marine planktonic Bacteria and Archaea. Nature. 2006;439(7078):847–50. 10.1038/nature04435 16482157

[79] Martin-Cuadrado A-B, Rodriguez-Valera F, Moreira D, Alba JC, Ivars-Martinez E, Henn MR, et al Hindsight in the relative abundance, metabolic potential and genome dynamics of uncultivated marine archaea from comparative metagenomic analyses of bathypelagic plankton of different oceanic regions. ISME J. 2008;2(8):865–86. 10.1038/ismej.2008.40 18463691

[80] Iverson V, Morris RM, Frazar CD, Berthiaume CT, Morales RL, Armbrust EV. Untangling Genomes from Metagenomes: Revealing an Uncultured Class of Marine Euryarchaeota. Science. 2012;335(6068). 10.1126/science.1212665 22301318

[81] Massana R, Delong EF, Pedrós-Alió C. A few cosmopolitan phylotypes dominate planktonic archaeal assemblages in widely different oceanic provinces. Appl Environ Microbiol. 2000;66(5):1777–87. 10788339 10.1128/aem.66.5.1777-1787.2000 PMC101412

[82] Delong EF. Oceans of Archaea. ASM News-American Society for Microbiology. 2003;69(10):503–503.

[83] Liu B, Ye G, Wang F, Bell R, Noakes J, Short T, et al Community Structure of Archaea in the Water Column above Gas Hydrates in the Gulf of Mexico. Geomicrobiol J. 2009;26(6):363–9.

[84] DeLong EF, Taylor LT, Marsh TL, Preston CM. Visualization and Enumeration of Marine Planktonic Archaea and Bacteria by Using Polyribonucleotide Probes and Fluorescent In Situ Hybridization. 1999;65(12):5554–63. 10584017 10.1128/aem.65.12.5554-5563.1999 PMC91757

[85] Swan BK, Martinez-Garcia M, Preston CM, Sczyrba A, Woyke T, Lamy D, et al Potential for Chemolithoautotrophy Among Ubiquitous Bacteria Lineages in the Dark Ocean. Science. 2011;333(6047). 10.1126/science.1203690 21885783

[86] Santoro AE, Dupont CL, Richter RA, Craig MT, Carini P, Mcilvin MR, et al Genomic and proteomic characterization of "Candidatus Nitrosopelagicus brevis": An ammonia-oxidizing archaeon from the open ocean. Proc Natl Acad Sci. 2015;112(4):1173–8. 10.1073/pnas.1416223112 25587132 PMC4313803

[87] Stieglmeier M, Mooshammer M, Kitzler B, Wanek W, Zechmeister-Boltenstern S, Richter A, et al Aerobic nitrous oxide production through N-nitrosating hybrid formation in ammonia-oxidizing archaea. ISME J. 2014;8(5):1135–46. 10.1038/ismej.2013.220 24401864 PMC3996696

[88] Naqvi SW a., Bange HW, Farías L, Monteiro PMS, Scranton MI, Zhang J. Marine hypoxia/anoxia as a source of CH4 and N2O. Biogeosciences. 2010;7(7):2159–90.

[89] Peng X, Fuchsman CA, Jayakumar A, Warner MJ, Devol AH, Ward BB. Revisiting nitrification in the Eastern Tropical South Pacific: A focus on controls. J Geophys Res Ocean. 2016;121(3):1667–84.

[90] Walker JT, Stow CA, Geron C. Nitrous Oxide Emissions from the Gulf of Mexico Hypoxic Zone. Environ Sci Technol. 2010;44(5):1617–23. 10.1021/es902058t 20131822

[91] Ravishankara AR, Daniel JS, Portmann RW. Nitrous oxide (N2O): the dominant ozone-depleting substance emitted in the 21st century. Science. 2009;326(5949):123–5. 10.1126/science.1176985 19713491

[92] Stocker BD, Roth R, Joos F, Spahni R, Steinacher M, Zaehle S, et al Multiple greenhouse-gas feedbacks from the land biosphere under future climate change scenarios. Nat Clim Chang. 2013;3(7):666–72.

[93] Pitcher A, Villanueva L, Hopmans EC, Schouten S, Reichart G-J, Sinninghe Damsté JS. Niche segregation of ammonia-oxidizing archaea and anammox bacteria in the Arabian Sea oxygen minimum zone. ISME J. 2011;5(12):1896–904. 10.1038/ismej.2011.60 21593795 PMC3223301

[94] Tolar BB, Wallsgrove NJ, Popp BN, Hollibaugh JT. Oxidation of urea-derived nitrogen by thaumarchaeota-dominated marine nitrifying communities. Environ Microbiol. 2016;00:1–13. 10.1111/1462-2920.13457 27422798

[95] Alonso-Sáez L, Waller AS, Mende DR, Bakker K, Farnelid H, Yager PL, et al Role for urea in nitrification by polar marine Archaea. Proc Natl Acad Sci. 2012 17989–94 p. 10.1073/pnas.1201914109 23027926 PMC3497816

[96] Church MJ, Delong EF, Ducklow HW, Karner MB, Preston CM, Karl DM. Abundance and distribution of planktonic Archaea and Bacteria in the waters west of the Antarctic Peninsula. 2003;48(5):1893–902.

[97] Sollai M, Hopmans EC, Schouten S, Keil RG, Sinninghe Damsté JS. Intact polar lipids of Thaumarchaeota and anammox bacteria as indicators of N cycling in the eastern tropical North Pacific oxygen-deficient zone. Biogeosciences. 2015;12:4725–37.

[98] Galand PE, Casamayor EO, Kirchman DL, Potvin M, Lovejoy C. Unique archaeal assemblages in the Arctic Ocean unveiled by massively parallel tag sequencing. ISME J. 2009;3(7):860–9. 10.1038/ismej.2009.23 19322244

[99] Horak REA, Qin W, Schauer AJ, Armbrust EV, Ingalls AE, Moffett JW, et al Ammonia oxidation kinetics and temperature sensitivity of a natural marine community dominated by Archaea. ISME J. 2013;7(10):2023–33. 10.1038/ismej.2013.75 23657360 PMC3965308

[100] Hollibaugh JT, Gifford SM, Moran MA, Ross MJ, Sharma S, Tolar BB. Seasonal variation in the metratranscriptomes of a Thaumarchaeota population from SE USA coastal waters. ISME J. 2014;8(3):685–98. 10.1038/ismej.2013.171 24132081 PMC3930313

[101] Tolar BB, Powers LC, Miller WL, Wallsgrove NJ, Popp BN, Hollibaugh JT. Ammonia Oxidation in the Ocean Can Be Inhibited by Nanomolar Concentrations of Hydrogen Peroxide. Front Mar Sci. 2016;3:237.

[102] Liu Q, Tolar BB, Ross MJ, Cheek JB, Sweeney CM, Wallsgrove NJ, et al Light and temperature control the seasonal distribution of thaumarchaeota in the South Atlantic bight. ISME J. 2018;12(6):1473–85. 10.1038/s41396-018-0066-4 29445129 PMC5956005

[103] Ulloa O, Canfield DE, DeLong EF, Letelier RM, Stewart FJ. Microbial oceanography of anoxic oxygen minimum zones. Proc Natl Acad Sci. 2012;109(40):15996–6003. 10.1073/pnas.1205009109 22967509 PMC3479542

[104] Capone DG, Hutchins DA. Microbial biogeochemistry of coastal upwelling regimes in a changing ocean. Nat Geosci. 2013;6(9):711–7.

[105] Altieri AH, Gedan KB. Climate change and dead zones. Glob Chang Biol. 2015;21(4):1395–406. 10.1111/gcb.12754 25385668

[106] Schmidtko S, Stramma L, Visbeck M. Decline in global oceanic oxygen content during the past five decades. Nature. 2017;542(7641):335–9. 10.1038/nature21399 28202958

