# Supplementary material for: Extent of the annual Gulf of Mexico hypoxic zone influences microbial community structure
Source: PLoS One. 2019 Apr 25;14(4):e0209055. doi: 10.1371/journal.pone.0209055 (PMC6483191; doi:10.1371/journal.pone.0209055)
Supplement: S1 File — Figure A. (A) Sample map of the 52 stations sampled during the Y14 nGOM shelfwide cruise, in which bottom water status is indicated (red circles indicates oxic stations while blue circles indicates hypoxic stations). (B) DO concentrations from the bottom samples collected. Figure B. (A) NMDS ordination of normalized 16S rRNA gene iTag sequence data for all samples collected in year 2014 grouped by surface and bottom. The seventeen bubble plots represent the same NMDS plot with normalized abundances of the OTUs that were statistically significantly different (Wilcoxon) between surface and bottom samples depicted by bubble size, where larger bubble size represents higher normalized abundances. The symbol * represents OTUs that were statistically significantly inversely correlated with dissolved oxygen (Spearman correlation; B-H corrected p-value ≤ 0.05). Figure C. (A) NMDS ordination of normalized 16S rRNA gene iTag sequence data for the all bottom samples collected in year 2014 where bubble size represents DO concentration. The other sixteen NMDS bubble plots represent the normalized abundances of the OTUs that were statistically significantly different (Wilcoxon) between hypoxic and oxic samples. Larger bubble size represents higher normalized abundances. The symbol * represents OTUs that were statistically significantly inversely correlated with dissolved oxygen while the other OTUs are significantly positively correlated with DO (Spearman correlation; B-H corrected p-value ≤ 0.05). Figure D. Plots of DO concentrations, relative abundances of Nitrosopumilus OTU4369009 16S rRNA genes (iTag), Thaumarchaeota 16S rRNA and amoA gene copy number/L (qPCR) for bottom water samples collected at the same sites in Y13 and Y14. Figure E. (A) NMDS ordination of normalized 16S rRNA gene iTag sequence data for the same samples collected in Y13 and Y14 with environmental variables, qPCR data and oligotype data shown as vectors. All environmental variables represented as vectors on [file pone.0209055.s001.pdf]

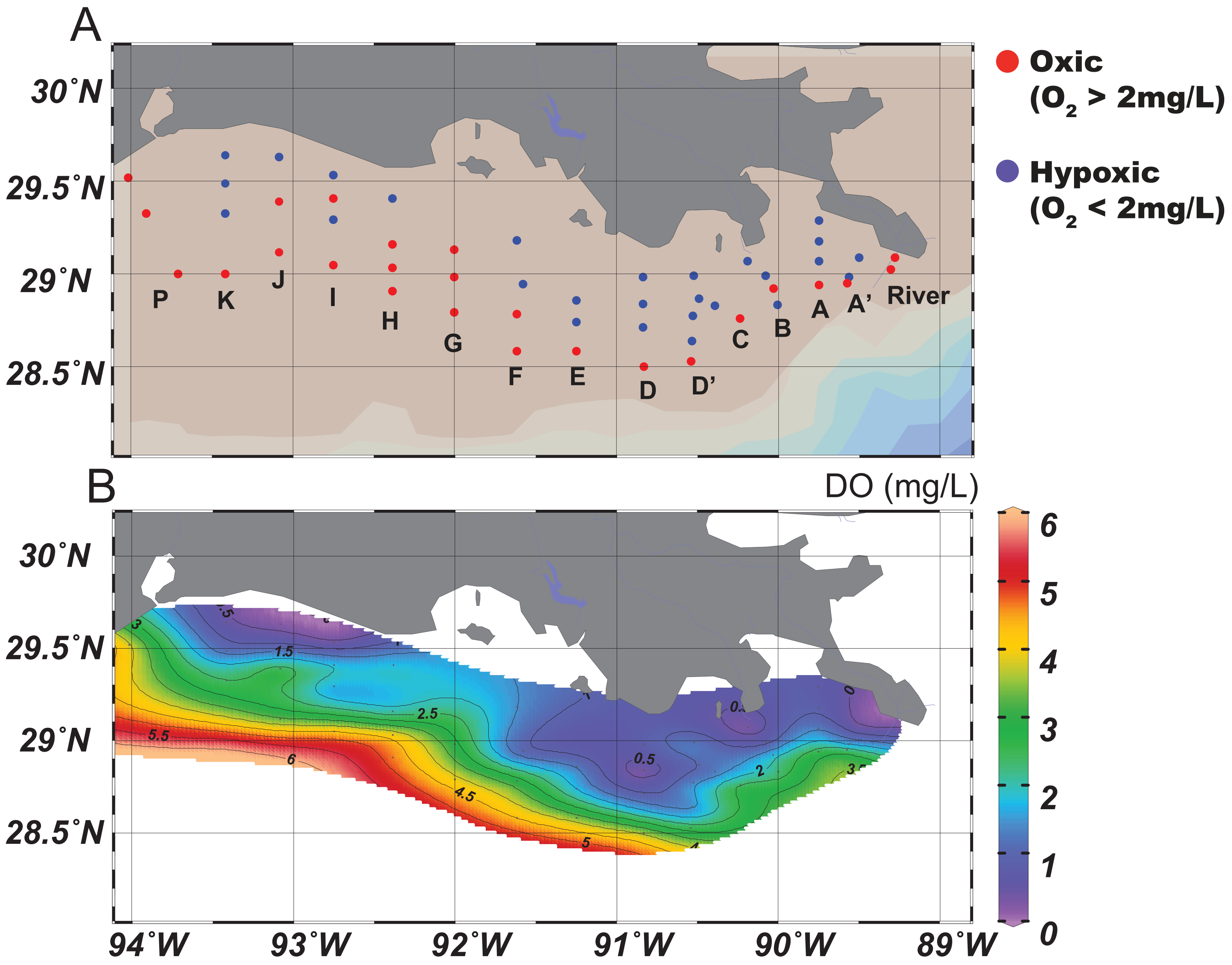

Figure A. (A) Sample map of the 52 stations sampled during the Y14 nGOM shelfwide cruise, in which bottom water status is indicated (red circles indicates oxic stations while blue circles indicates hypoxic stations). (B) DO concentrations from the bottom samples collected.

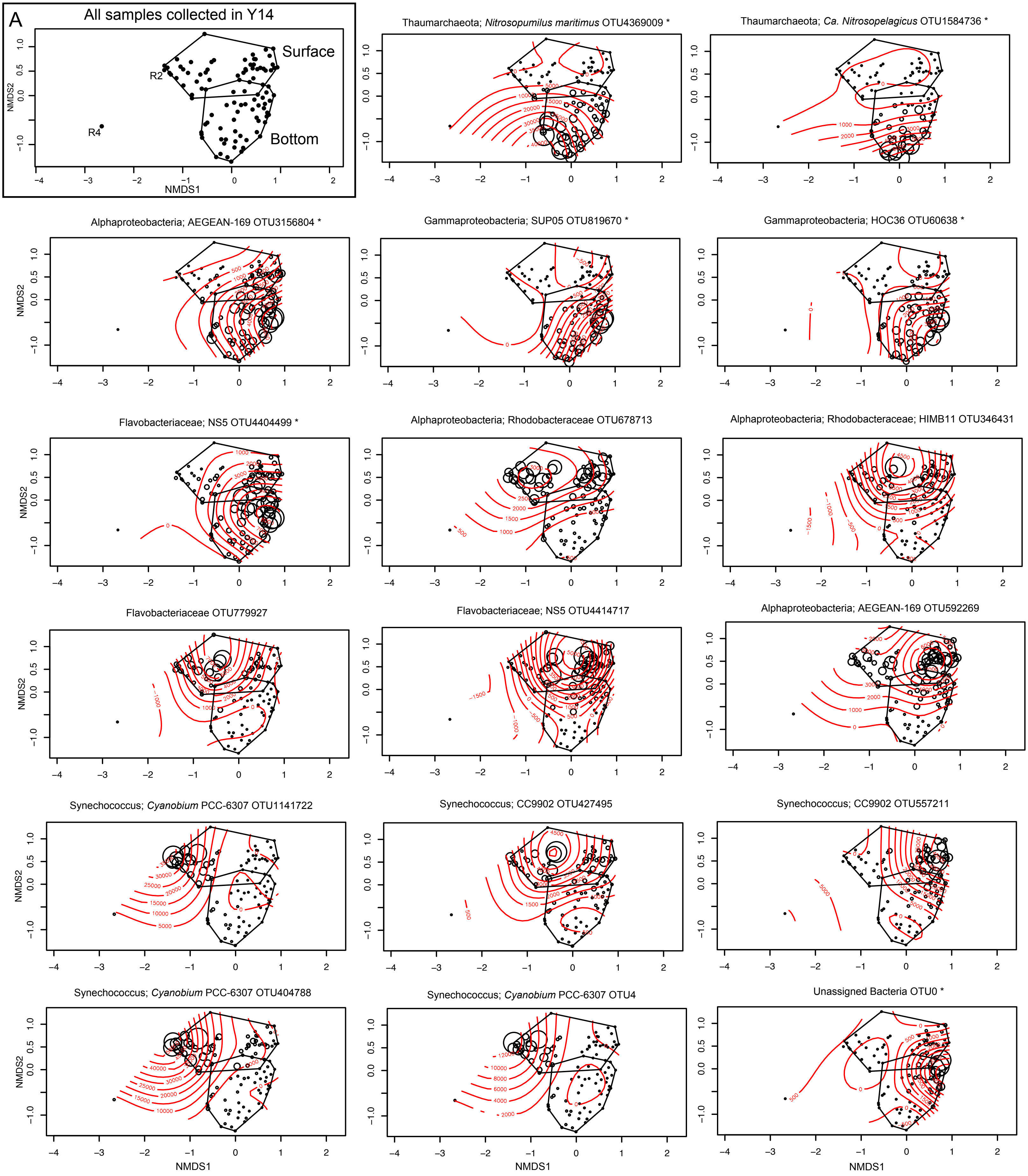

Figure B. (A) NMDS ordination of normalized 16S rRNA gene iTag sequence data for all samples collected in year 2014 grouped by surface and bottom. The seventeen bubble plots represent the same NMDS plot with normalized abundances of the OTUs that were statistically significantly different (Wilcoxon) between surface and bottom samples depicted by bubble size, where larger bubble size represents higher normalized abundances. The symbol * represents OTUs that were statistically significantly inversely correlated with dissolved oxygen (Spearman correlation; B-H corrected p-value ≤ 0.05).

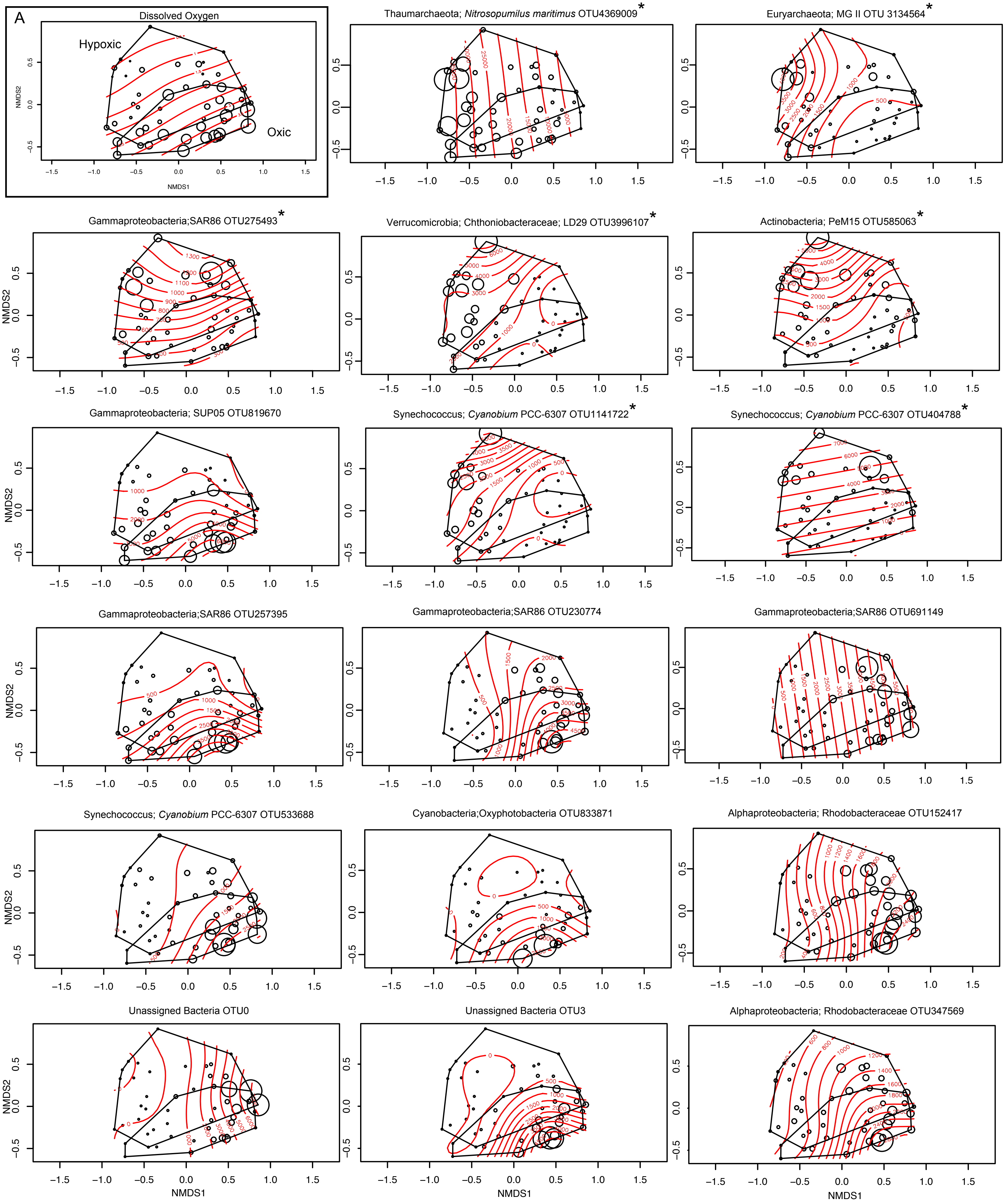

Figure C. (A) NMDS ordination of normalized 16S rRNA gene iTag sequence data for the all bottom samples collected in year 2014 where bubble size represents DO concentration. The other sixteen NMDS bubble plots represent the normalized abundances of the OTUs that were statistically significantly different (Wilcoxon) between hypoxic and oxic samples. Larger bubble size represents higher normalized abundances. The symbol * represents OTUs that were statistically significantly inversely correlated with dissolved oxygen while the other OTUs are significantly positively correlated with DO (Spearman correlation; B-H corrected p-value ≤ 0.05).

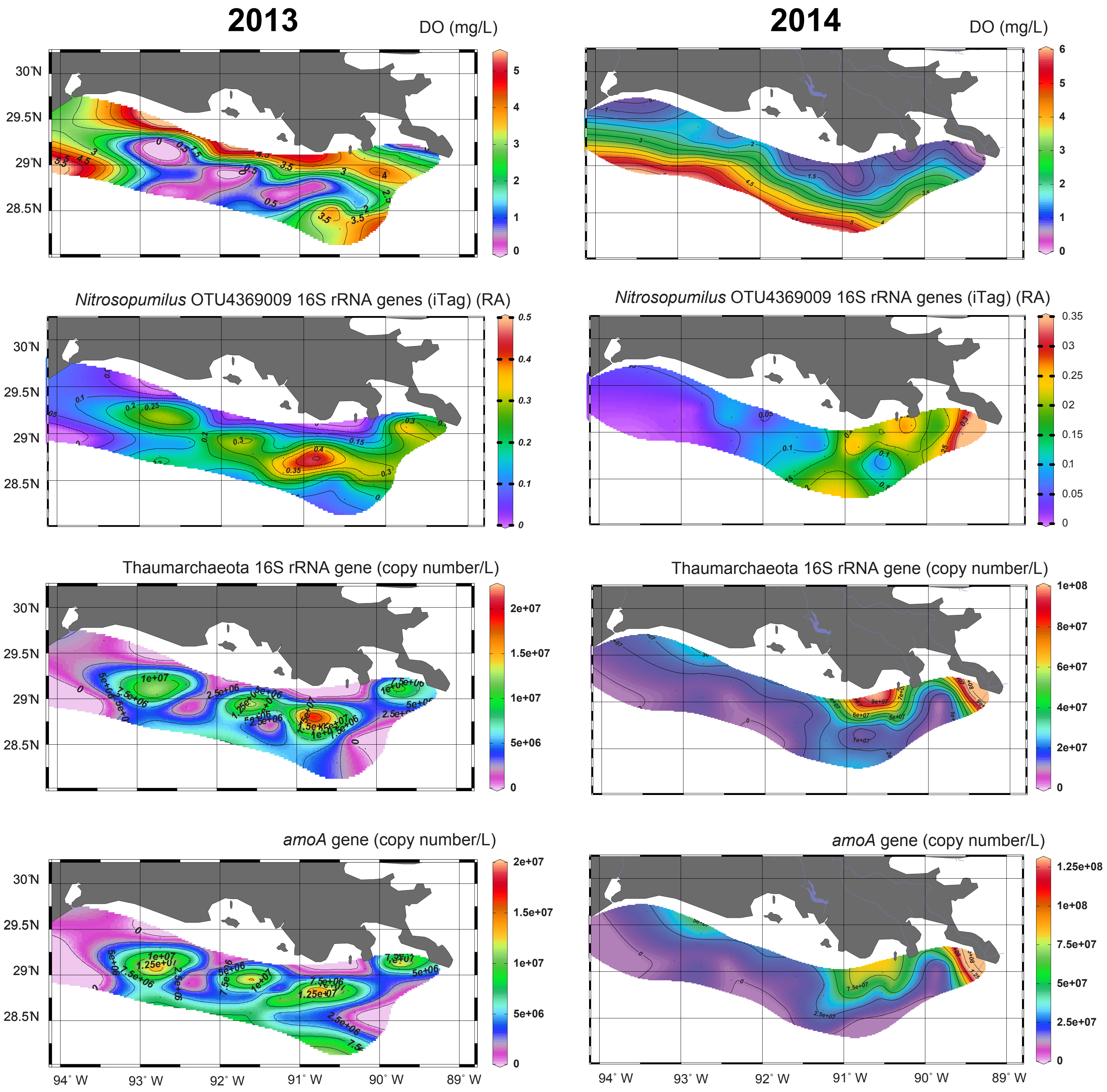

Figure D. Plots of DO concentrations, relative abundances of *Nitrosopumilus* OTU4369009 16S rRNA genes (iTag), Thaumarchaeota 16S rRNA and *amoA* gene copy number/L (qPCR) for bottom water samples collected at the same sites in Y13 and Y14.

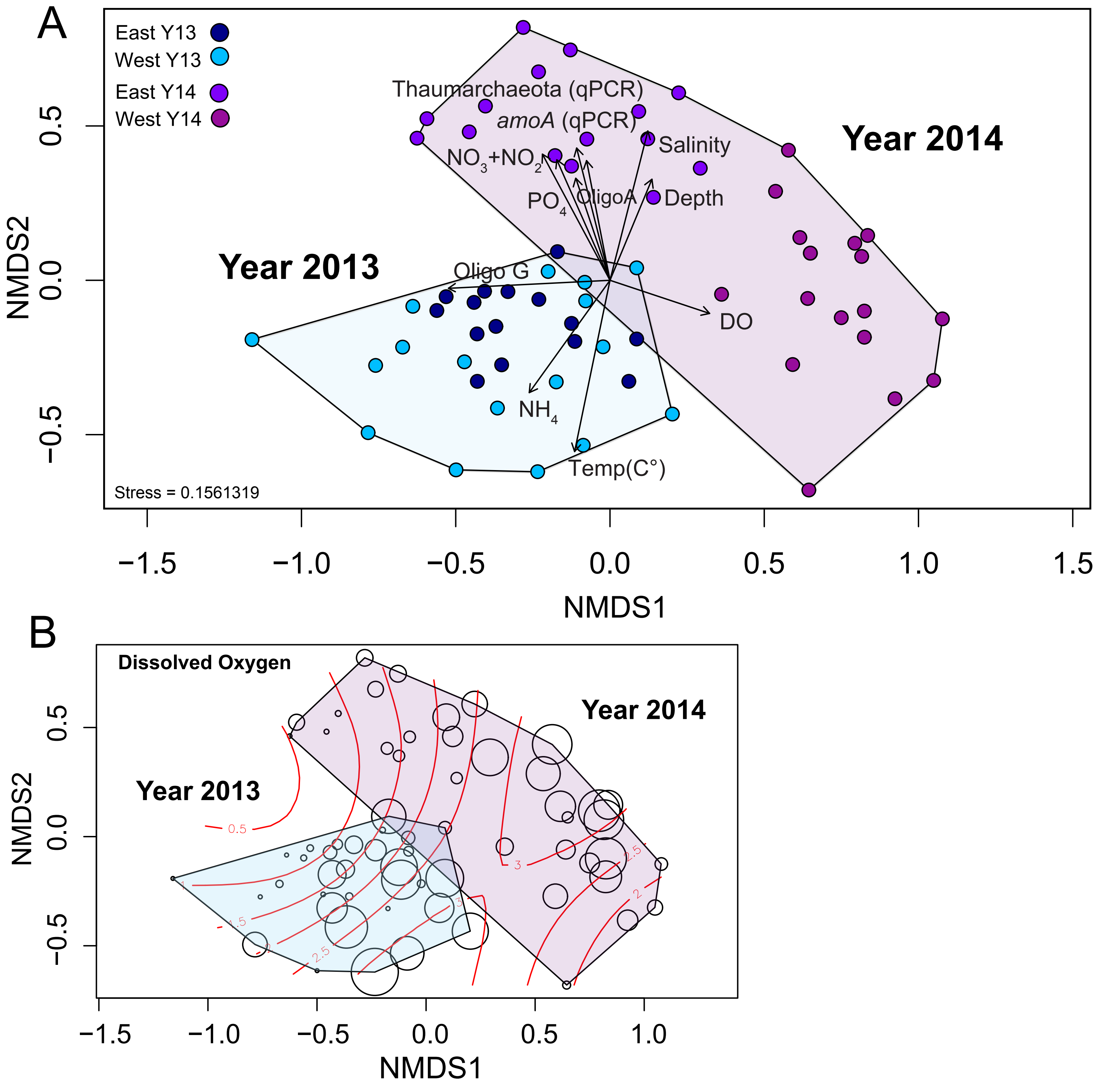

Figure E. (A) NMDS ordination of normalized 16S rRNA gene iTag sequence data for the same samples collected in Y13 and Y14 with environmental variables, qPCR data and oligotype data shown as vectors. All environmental variables represented as vectors on the NMDS were significantly correlated (corrected p-value ≤ 0.05) with an NMDS axis. **(B)** The same NMDS ordination depicting oxygen concentration (DO) as bubble size e.g. larger bubbles indicate higher DO concentrations.

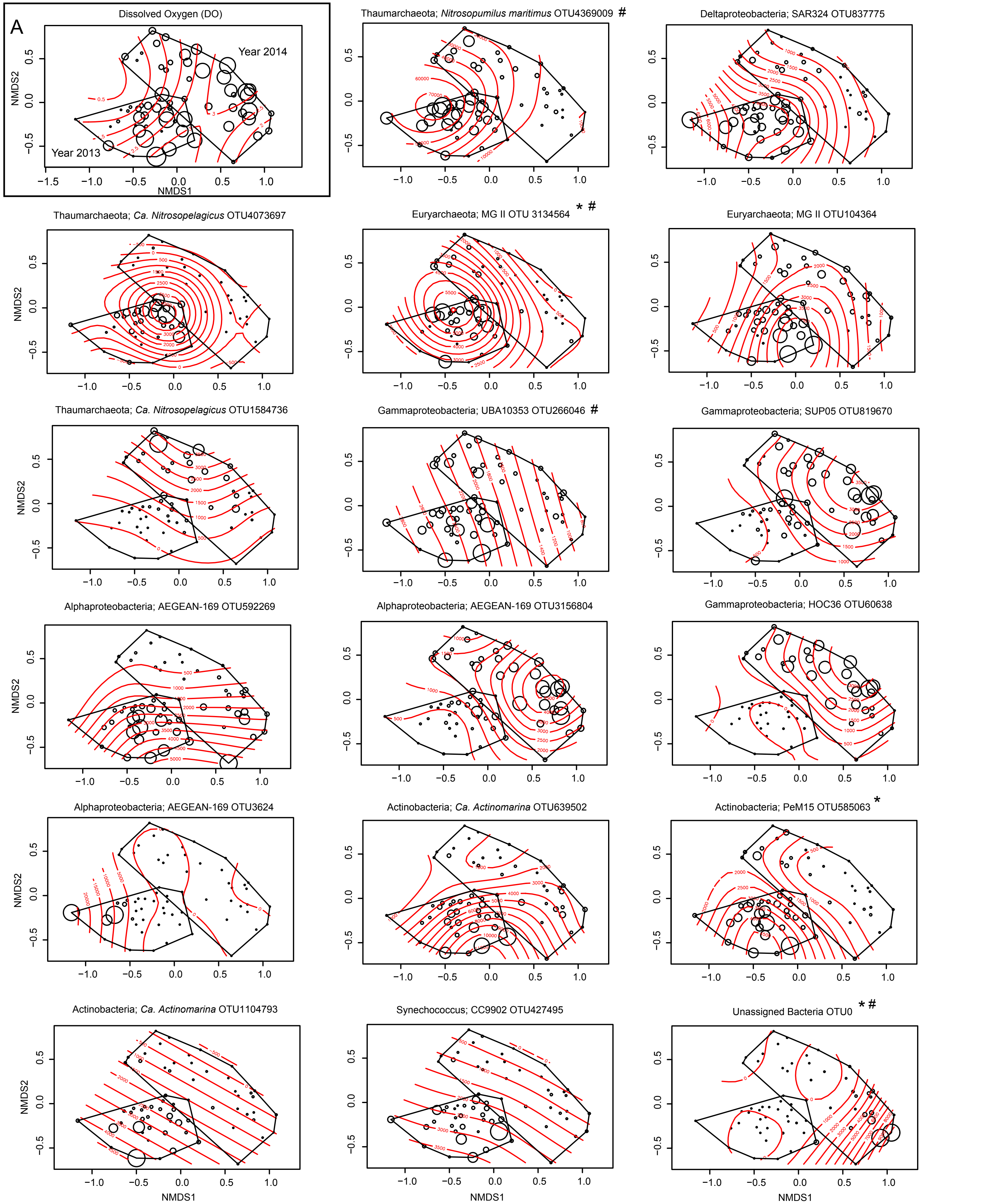

Figure F. **(A)** NMDS ordination of normalized 16S rRNA gene iTag sequence data for the same samples collected in Y13 and Y14 where bubble size depicts oxygen concentration. The other seventeen NMDS bubble plots represent the normalized abundances of the OTUs that were statistically significantly different (Wilcoxon) between the years where larger bubble size represents higher normalized abundances. The symbol ***** represents OTUs that were statistically significantly inversely correlated with DO in Y14, and the symbol **#** represents a significant inverse correlation with DO in Y13 (Spearman correlation; B-H corrected p-value ≤ 0.05).

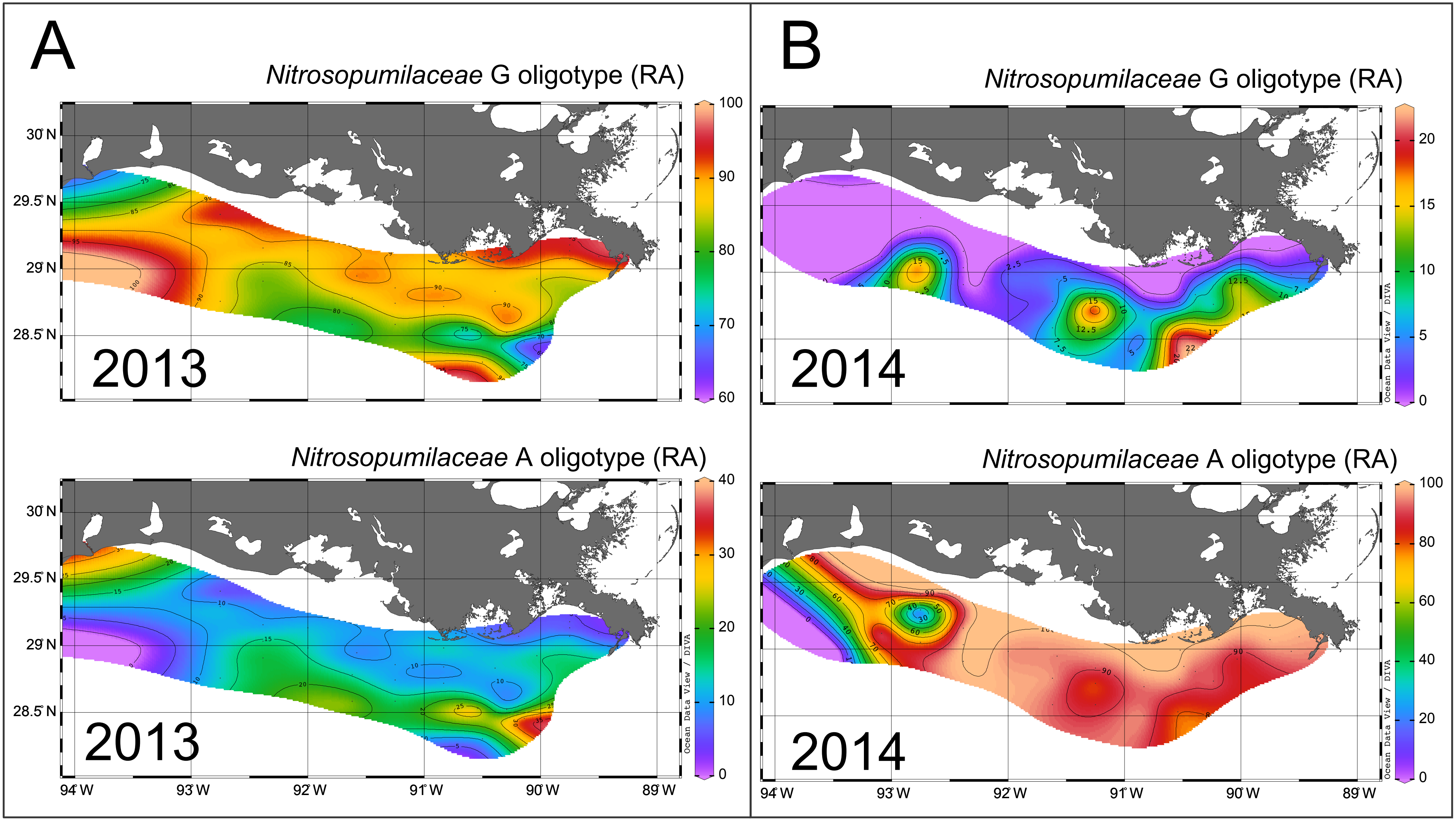

Figure G. Plots of (A) Oligotype data for *Nitrosopumilaceae* G and A oligotypes for the same samples collected in Y13 (B) Oligotype data for *Nitrosopumilaceae* G and A oligotypes for the same samples collected in Y14.
